# S100A4 enhances protumor macrophage polarization by control of PPAR-γ-dependent induction of fatty acid oxidation

**DOI:** 10.1136/jitc-2021-002548

**Published:** 2021-06-18

**Authors:** Shuangqing Liu, Huilei Zhang, Yanan Li, Yana Zhang, Yangyang Bian, Yanqiong Zeng, Xiaohan Yao, Jiajia Wan, Xu Chen, Jianru Li, Zhaoqing Wang, Zhihai Qin

**Affiliations:** 1 Key Laboratory of Protein and Peptide Pharmaceuticals, Institute of Biophysics, Chinese Academy of Sciences, Beijing, China; 2 University of Chinese Academy of Sciences, Beijing, China; 3 Medical Research Center, The First Affiliated Hospital of Zhengzhou University, Zhengzhou, China; 4 School of Basic Medical, Southwest Medical University, Luzhou, China

**Keywords:** immunity, innate, immunomodulation, tumor microenvironment, macrophages, metabolic networks and pathways

## Abstract

**Background:**

The peroxisome proliferator-activated receptor γ (PPAR-γ)-dependent upregulation of fatty acid oxidation (FAO) mediates protumor (also known as M2-like) polarization of tumor-associated macrophages (TAMs). However, upstream factors determining PPAR-γ upregulation in TAM protumor polarization are not fully identified. S100A4 plays crucial roles in promotion of cancer malignancy and mitochondrial metabolism. The fact that macrophage-derived S100A4 is major source of extracellular S100A4 suggests that macrophages contain a high abundance of intracellular S100A4. However, whether intracellular S100A4 in macrophages also contributes to cancer malignancy by enabling TAMs to acquire M2-like protumor activity remains unknown.

**Methods:**

Growth of tumor cells was evaluated in murine tumor models. TAMs were isolated from the tumor grafts in whole-body S100A4-knockout (KO), macrophage-specific S100A4-KO and transgenic S100A4^WT−EGFP^ mice (expressing enhanced green fluorescent protein (EGFP) under the control of the S100A4 promoter). In vitro induction of macrophage M2 polarization was conducted by interleukin 4 (IL-4) stimulation. RNA-sequencing, real-time quantitative PCR, flow cytometry, western blotting, immunofluorescence staining and mass spectrometry were used to determine macrophage phenotype. Exogenous and endogenous FAO, FA uptake and measurement of lipid content were used to analyze macrophage metabolism.

**Results:**

TAMs contain two subsets based on whether they express S100A4 or not and that S100A4^+^ subsets display protumor phenotypes. S100A4 can be induced by IL-4, an M2 activator of macrophage polarization. Mechanistically, S100A4 controls the upregulation of PPAR-γ, a transcription factor required for FAO induction during TAM protumor polarization. In S100A4^+^ TAMs, PPAR-γ mainly upregulates CD36, a FA transporter, to enhance FA absorption as well as FAO. In contrast, S100A4-deficient TAMs exhibited decreased protumor activity because of failure in PPAR-γ upregulation-dependent FAO induction.

**Conclusions:**

We find that macrophagic S100A4 enhances protumor macrophage polarization as a determinant of PPAR-γ-dependent FAO induction. Accordingly, our findings provide an insight into the general mechanisms of TAM polarization toward protumor phenotypes. Therefore, our results strongly suggest that targeting macrophagic S100A4 may be a potential strategy to prevent TAMs from re-differentiation toward a protumor phenotype.

## Background

Tumor-associated macrophages (TAMs), primarily originated from blood monocytes, are continuously recruited to the tumor mass to constitute a supportive tumor microenvironment (TME) during tumor development.^1 2^ Due to their high plasticity and diversity, the infiltrated TAMs can undergo coordinated changes in gene expression and metabolic programming in response to TME cues such as Th2 cytokine interleukin 4 (IL-4), which alternatively activates them toward a protumor (also known as M2-like) phenotype with anti-inflammatory and immunosuppressive properties.^3 4^ These protumor TAMs facilitate persistent tumor proliferation and metastasis, contributing to tumor progression, therapeutic resistance and poor survival prognosis.^5^ However, the mechanisms underlying how antitumor (also known as M1-like) TAMs convert into protumor TAMs remain unclear. Nevertheless, how TAMs are generated and modulated remains to be further deeply explored.

One well-known mechanism involved in polarization of TAMs is cellular metabolism, which plays a key role in the control of TAM plasticity and diversity.^6^ Protumoral TAMs prefer to use mitochondria-dependent fatty acid oxidation (FAO) as their energy supply.^7^ Correspondingly, inhibition of FAO in TAMs impedes alternative polarization of TAMs toward protumor phenotype and inhibits tumor growth.^8–12^ Mechanistic analyses indicate that such metabolic reprogramming of FAO upregulation in TAMs is dependent on the induction of peroxisome proliferator-activated receptor γ (PPAR-γ), a transcriptional factor, which is required for TAM protumoral polarization.^9 13^ The essential role of PPAR-γ can be partially attributed to the upregulation its target gene, the FA transporter CD36, during the process of FAO induction-regulated TAM protumor polarization.^14^ However, upstream factors determining PPAR-γ activation in TAM protumor polarization are not fully identified.

S100A4, also known as FSP1, MTS1 or metastasin, is a well-established metastasis-promoting oncoprotein with potent protumor activity.^15 16^ It belongs to the S100 superfamily Ca^2+^‐binding proteins and is not only expressed by cancer cells but also by various stromal cells.^17^ Like the cancerous S100A4, the stromal counterparts also play essential roles in promotion of cancer malignancy.^18 19^ However, the specific S100A4^+^ stromal cell types that are involved in tumor progression have not been well characterized.^20^

Our previous studies have shown that macrophages are a major source of soluble S100A4 in liver and lung fibrosis,^21 22^ suggesting that macrophages contain a high abundance of S100A4. Clinical studies indicate that S100A4 is preferentially expressed by macrophages, activated lymphocytes and fibroblasts in the TME, rather than by the tumor cells themselves.^17^ A recent analysis of the increased cell lineages in injured livers showed that S100A4^+^ cells expressed markers of the myeloid-monocytic lineage, including F4/80 and CD11b, indicating that S100A4 is a reliable marker of a specific subset of macrophages.^23^ Considering these findings, the present study investigated, in the context of tumor pathology, whether S100A4 may also contribute to cancer progression by enabling TAMs to acquire M2-like protumor activity.

## Methods

### Mice, care and use

The C57BL/6 background *s100a4* floxed mice were produced from GemPharmatech (Nanjing, Jiangsu, China) and then were backcrossed with wild type (WT) C57BL/6 mice for at least six generations. The *s100a4* floxed mice were crossed with lysozyme M (LysM)-Cre mice (B6/JNju -LysM^em1Cin (iCre)^/Nju, T003822) to produce macrophage-specific conditional *s100a4* knockout (KO) (*s100a4*^flox/flox^ LysM-Cre, called S100A4^M−KO^) mice. The generation of the C57BL/6 background whole-body S100A4-KO (named S100A4^KO^) mice and transgenic S100A4^WT−EGFP^ mice (expressing EGFP under the control of the S100A4 promoter), was described as the previous report.^21^ The BALB/c background S100A4-thymidine kinase (TK) transgenic (named S100A4^TK+^) mice were obtained from Dr. Eric G. Neilson’s lab (Northwestern University, Feinberg School of Medicine).^24^ These mice express a truncated TK gene under the control of the *s100a4* promoter. Treatment with ganciclovir (GCV) can selectively ablate dividing (proliferating) cells. All mice were bred and maintained under specific pathogen-free conditions in the animal facilities, and all animal testing and research were performed with sex-matched and age-matched mice.

### Measurement of exogenous and endogenous FAO

The respiration changes, caused by utilization of exogenous FAs, endogenous FAs, or uncoupling by FAs, were simultaneously measured using Seahorse XF Cell Mito Stress Test Kit (103015–100, Agilent, Palo Alto, California, USA) that works with the Seahorse XF analyzer and can simultaneously measure oxidation of exogenous and endogenous FA. The tested S100A4^WT^ and S100A4^KO^ Raw264.7 cells (10,000/well) were cultured on the XF^96^ cell culture microplate (102601–100, Agilent, Palo Alto, California, USA) and were stimulated with or without IL-4 (20 ng/mL) for 36 hours. Then the cell culture medium was changed to substrate-limited medium and cells were further cultured in this medium for 7 hours. The cells were washed with FAO assay medium once and incubated for 30 min in 37 °C cell incubator without CO_2_. The following test steps were according to manufacturer’s protocol. Oxygen consumption rate (OCR) was automatically calculated by the Seahorse XF-96 software in response to 2.5 µg/mL oligomycin, 0.8 µM carbonyl cyanide 4-(trifluoromethoxy) phenylhydrazone (FCCP), 2 µM rotenone plus 4 µM antimycin A, and 40 µM etomoxir (ETO, Sigma-Aldrich, Darmstadt, Germany).

### FA uptake assay

FA uptake was measured using a free FA uptake assay fluorometric kit (ab176768, Abcam, Cambridge, UK). Cells (1×10^5^/well) were resuspended in Hank’s balanced salt solution and incubated for 30 min at 37°C in a CO_2_ incubator, followed by the addition of the fluorescent FA probe. After 1-hour incubation, fluorescence levels were determined on fluorescence microplate reader with a bottom-read mode at Ex/Em=485/515 nm or FITC channel. For kinetic reading: checking the fluorescence intensity immediately at 20 s interval for 30–60 min. For endpoint reading: checking the fluorescence intensity at the end of the 30–60 min incubation. The data were acquired by fluorescence microplate reader for kinetic reading. ‘Reads’ meant the number of times that fluorescence value can be read.

### Lipid droplet staining with Nile red

Cells were cultured with medium supplemented with oleate (0.2 mM, Sigma, Darmstadt, Germany) in the presence or absence of CD36 inhibitor, sulfo-N-succinimidyl oleate Na (SSO, 25 µM, Sigma) for indicated time. The cells were then washed with phosphate buffered saline (PBS) and stained with Nile red (1:5000, Invitrogen, Carlsbad, California, USA) for 30 min at 37°C. The quantification of Nile red content was measured by flow cytometry.

### RNA-sequencing analysis

Murine TAMs that isolated from the E0771 breast cancer cell-bearing S100A4^WT-EGFP^ mice were sorted by flow cytometry into two subset populations: CD45^+^F4/80^+^CD11b^+^EGFP^+^ and CD45^+^F4/80^+^CD11b^+^EGFP^−^. The sorted cells were applied for total RNA extraction with RNeasy Mini Kit (QIAGEN, Dusseldorf, Germany) and subjected to RNA-sequencing (RNA-seq) analysis by Novogene (Beijing, China). The raw transcriptomic reads were mapped to a reference genome (GRCm38/mm10) by using Bowtie. Gene expression levels were quantified by the RSEM software package. Significantly differentially expressed genes were acquired by setting padj <0.05, and log_2_ fold change >0.0.

### Statistical analysis

Statistical analysis was performed using GraphPad Prism V.7.0. Data are shown as mean±SE. Comparisons between two groups were calculated using unpaired Student’s *t*-test or unpaired nonparametric Mann Whitney test and comparisons between two groups at multiple time points were calculated by two-way analysis of variance (ANOVA) Sidak’s multiple comparisons. Comparisons of more than two groups were calculated using one-way ANOVA Tukey’s multiple comparisons. In figures, asterisks denote statistical significance (∗, *P*<0.05; ∗∗, *P*<0.01; ∗∗∗, *P*<0.001).

## Results

### S100A4^+^ TAMs exhibit protumor phenotype and function

**Figure 1:**
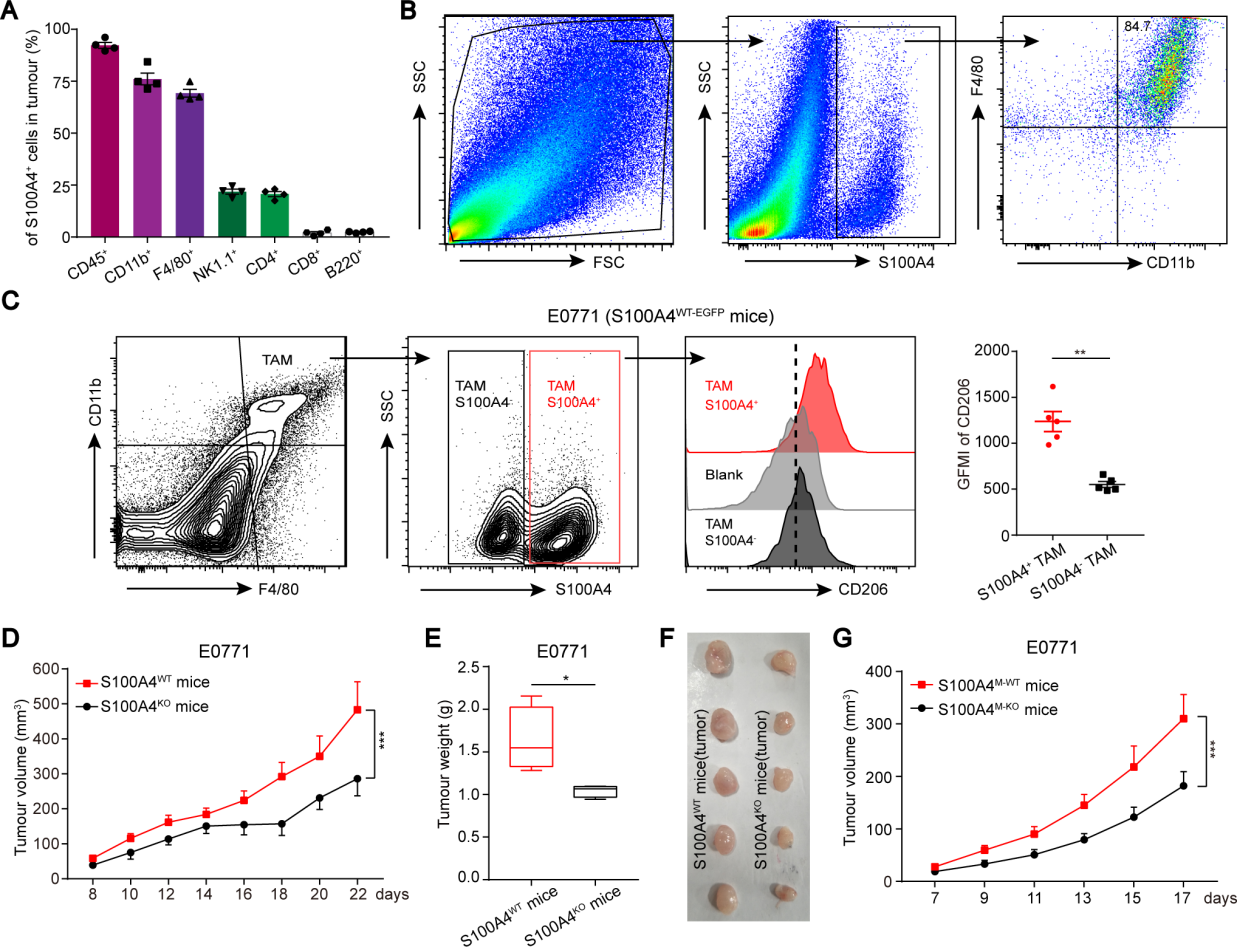
S100A4^*+*^ TAMs possess protumor activity. (A) Flow cytometric analysis of the frequency of immune cell populations in tumor-infiltrating S100A4^+^ cells of four mice. (B) Flow cytometric analysis of TAMs frequency in S100A4^WT-EGFP^ cells by double staining with CD11b and F4/80. (C) Flow cytometric analysis of the frequency of S100A4^+^ TAMs (CD11b^+^/F4/80^+^) and the fluorescence intensity of CD206 in S100A4^*+*^ and S100A4^−^ TAMs. (D–F) Growth of tumor grafts was monitored over time after the initial injection of E0771 breast cancer cells into S100A4^WT^ or S100A4^KO^ female mice (n≥5). tumor weight and representative pictures of tumor grafts excised at the end of the experiment. (G) Growth of tumor grafts was monitored over time after initial inoculation of E0771 breast cancer cells into S100A4^M-WT^ or S100A4^M-KO^ female mice (n≥8). Data are presented as mean±SE and were analyzed by unpaired non-parametric Mann-Whitney U test in C, E or two-way ANOVA Sidak's multiple comparisons in D, G. The data are from one representative experiment of more than three independent experiments (B–G). *P<0.05, **P<0.01, ***P<0.001. SSC, side scatter; ANOVA, analysis of variance; GMFI, geometric mean fluorescence intensity; KO, knockout; TAM, tumor-associated macrophage; WT, wild type.

To explore whether S100A4 is expressed on TAMs, we injected E0771 breast cancer cells into the mammary pads of female S100A4 WT (S100A4^WT-EGFP^) reporter mice, expressing EGFP under the control of the *s100a4* gene promoter.^21 25^ Then, we collected TAMs and analyzed the immune contexture of S100A4^WT-EGFP^ cells inside the tumor grafts using different markers for immune cell types. The majority of the tumor-infiltrating S100A4^WT-EGFP^ immune cells were CD11b^+^ or F4/80^+^ myeloid cells, whereas lymphocytes, including T cells, B cells and NK cells, were in the minority (figure 1A and online supplemental figure 1A). Because CD11b^+^ and F4/80^+^ double positive cells are macrophages, we stained S100A4^+^ cells with both CD11b and F4/80 and found that TAMs with CD11b^+^ and F4/80^+^ accounted for major components in S100A4^+^ subsets (figure 1B). To remove the potential influence of gender on the above observations, we established another tumor model in which male S100A4^WT-EGFP^ mice were implanted with MCA205 fibrosarcoma cells. Similar results were observed in this tumor graft to those in the E0771 model (online supplemental figure 1B).

10.1136/jitc-2021-002548.supp1 Supplementary data

We subsequently used CD206, a well-established marker of M2 macrophages, to distinguish M2-like protumor TAMs in the immune contexture from the E0771 tumor grafts. The data showed that the isolated TAMs could apparently be divided into two phenotypic subsets: S100A4^+^ TAMs and S100A4^−^ TAMs (figure 1C). The significantly increased CD206 expression in S100A4^+^ TAMs compared with S100A4^−^ TAMs indicated that S100A4 was mainly expressed in TAMs with protumor phenotype.

Next, we explored the roles of S100A4^+^ TAMs in tumor development using two S100A4-KO mouse models. First, whole-body S100A4 KO (S100A4^KO^) and control WT S100A4^WT^ mice were implanted with E0771 and MCA205 cells. We observed that systemic S100A4 deficiency markedly impeded tumor growth and caused a significant decrease in tumor weight but did not affect the body weight of the mice (figure 1D-F and online supplemental figure 1C, D). Second, to investigate the association of macrophagic S100A4 with tumor development, we developed a macrophage-specific S100A4-KO (S100A4^M-KO^) murine model. E0771 cells were implanted into S100A4^M-KO^ and control S100A4^M-WT^ female mice (online supplemental figure 2A−C). Significantly inhibited tumor development was observed in the animals with macrophagic-specific S100A4 deletion compared with control animals (figure 1G). These results clearly indicated that S100A4^+^ TAMs have protumor functions and S100A4 may be involved in phenotype switching of TAMs.

### Macrophagic S100A4 enhances TAM protumor polarization

**Figure 2:**
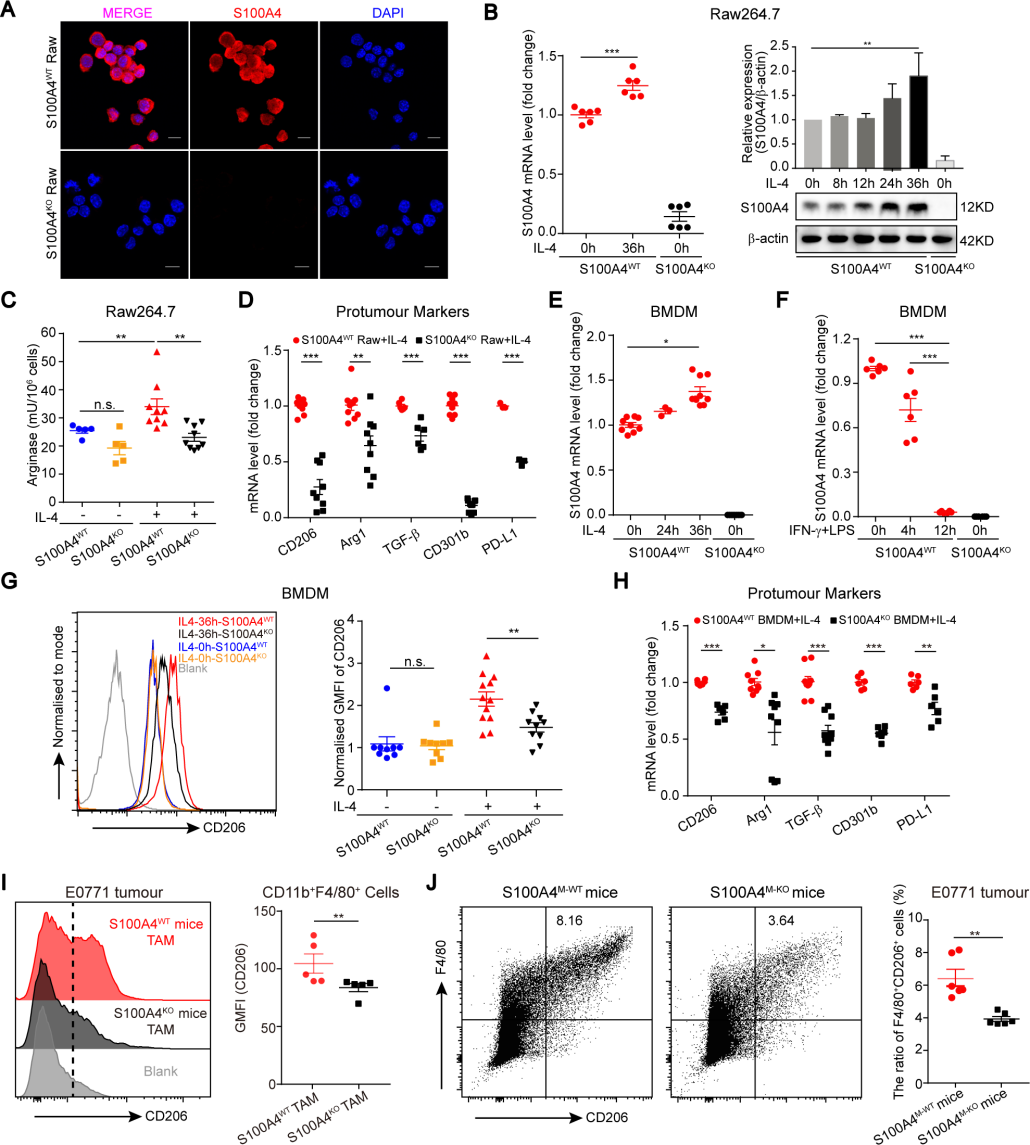
Macrophagic deficiency of S100A4 impairs TAM alternative activation. (A–D) S100A4^WT^ or S100A4^KO^ Raw264.7 cells were activated with IL-4 (20 ng/mL). Nonactivated macrophages were used as controls. A Lack of S100A4 expression was analyzed by immunofluorescence in S100A4^WT^ or S100A4^KO^ Raw264.7 cells (middle). The immune complexes were detected with a secondary antibody conjugated to Alexa Fluor 555 (red). DNA was stained with DAPI (blue). Scale bars, 10 μm. Expression of S100A4 mRNA and protein was analyzed by q-PCR (left in B) and immunoblotting (right in B), respectively. Band densities (mean±SE) for S100A4 were measured in at least three independent immunoblots and normalized to those of β-actin (loading control). Arginase activity was assessed with or without 36-hours activation with IL-4 in (C). Expression of M2 hallmarks was assessed by q-PCR after 36-hours activation with IL-4 in D. The values were normalized with that in S100A4^WT^ Raw264.7 cells with IL-4 stimulation. (E–H) S100A4^WT^ or S100A4^KO^ BMDMs were treated with IL-4 (20 ng/mL, E) or IFN-γ (20 ng/mL) in combination with LPS (100 ng/mL, F). S100A4 mRNA expression was analyzed by q-PCR in E, F. The values were normalized with that in S100A4^WT^ BMDMs without stimulation. After 36-hours activation with IL-4, the expression of CD206 was examined and quantified by flow cytometry in G and expression of M2 hallmarks was assessed by q-PCR in H. (I, J) Flow cytometric analysis of CD206 expression in TAMs and the proportion of CD206^+^ TAMs (I) in the tumor grafts from E0771 breast cancer cell-bearing S100A4^WT^ and S100A4^KO^ female mice (n=5) or (J) S100A4^M-WT^ and S100A4^M-KO^ female mice (n=6). Data are presented as mean±SE and were analyzed by unpaired Student’s t-test in B–G, and H and by unpaired nonparametric Mann-Whitney U test in I, J. The data are from one representative experiment of more than three independent experiments (A–C, I, J). *P<0.05, **P<0.01, ***P<0.001, BMDMs, bone marrow-derived macrophages; GMFI, geometric mean fluorescence intensity; IFN-γ, interferon-γ; IL-4, interleukin 4; KO, knockout; n.s., not significant; TAM, tumor-associated macrophage; WT, wild type; DAPI, 4',6-diamidino-2-phenylindole.

Next, we investigated whether S100A4 is involved in macrophage polarization. To assess this, we specifically knocked out S100A4 in Raw264.7 cells using CRISPR/Cas9 technology and established an S100A4 KO (S100A4^KO^) Raw264.7 cell line. The lack of S100A4 expression in S100A4^KO^ Raw264.7 cells was confirmed by immunofluorescent staining (figure 2A and online supplemental figure 3A), PCR and immunoblotting (online supplemental figure 3B, C). The number 1 clone among three cell lines with decreased expression of CD206 (online supplemental figure 3D) was selected for further experiments. We also noted that there was no difference in the proliferating activity of S100A4^WT^ and S100A4^KO^ Raw264.7 cells (online supplemental figure 3E).

Although S100A4 is an inducible protein that responds to extracellular signals such as transforming growth factor-β (TGF-β), Wnt or hypoxia,^26–28^ it is unclear whether M1 or M2 activators can induce S100A4 expression. To this end, we treated S100A4^WT^ Raw264.7 cells with IL-4 or interferon-γ (IFN-γ) in combination with LPS, which are the well-known activators of M2 and M1 macrophage phenotypes, respectively. In these S100A4^WT^ Raw264.7 cells there was a robust, time-dependent increase in S100A4 mRNA and protein expression in response to IL-4 (figure 2B and online supplemental Table 1), whereas S100A4 expression decreased in response to IFN-γ/LPS with (online supplemental figure 3F).

We next wondered whether S100A4 deficiency influences the IL-4−induced macrophage polarization of Raw264.7 cells. Because increased arginase activity is a well-established index of TAM M2-like polarization, we first examined this index in S100A4^WT^ and S100A4^KO^ Raw264.7 cells. In the presence of IL-4, S100A4-KO in Raw264.7 cells dramatically impaired arginase activities compared with WT cells. In contrast, in the absence of IL-4, the changes of arginase activities between S100A4^WT^ and S100A4^KO^ Raw264.7 cells are marginal (figure 2C). Based on the above data, we further analyzed the changes of other M2-markers between S100A4^WT^ and S100A4^KO^ Raw264.7 cells only in the presence of IL-4 without including no IL-4 treatment control. Consistently, we found that S100A4-KO significantly inhibited the expression of the examined genes, including *cd206*, *arginase*, *tgf-β*, *cd301b* and *pd-l1*, the values were normalized with S100A4^WT^ Raw264.7 cells (figure 2D).

To further confirm the above observations, we established S100A4^WT^ and S100A4^KO^ bone marrow-derived macrophages (BMDMs), which were isolated from S100A4^WT^ and S100A4^KO^ mice and were stimulated in vitro by macrophage colony stimulating factor (online supplemental figure 4A). A similar pattern of S100A4 expression stimulated by IL-4 or IFN-γ/LPS to that found in S100A4^WT^ Raw264.7 cells was also observed in S100A4^WT^ BMDMs (figure 2E, F). Using established BMDMs from S100A4^WT−EGFP^ mice, we observed that the majority of BMDMs expressed S100A4 with high abundance (online supplemental figure 4B). In S100A4^WT^ and S100A4^KO^ BMDMs, we first examined the expression of CD206 by flow cytometry analysis. Accordingly, in the presence of IL-4, S100A4 deficiency in BMDMs also resulted in a significant decrease in the CD206 expression. In the absence of IL-4, the influence of S100A4 deficiency on the CD206 expression is limited (figure 2G). In addition, the expressions of other M2 markers were significantly inhibited in S100A4^KO^ BMDMs compared with S100A4^WT^ BMDM (figure 2H). Furthermore, we also checked the major histocompatibility complex class-II (MHC-II) expression in IFN-γ/LPS-stimulated S100A4^WT^ and S100A4^KO^ BMDMs. No difference in MHC-II expression was observed between these two cells (online supplemental figure 4C). Altogether, these results provide direct evidence that S100A4 is required for TAM alternative polarization towards the M2 phenotype.

Because IL-4-activated macrophages cannot fully represent the characteristics of TME-educated TAMs, we further explored the in vivo roles of S100A4 in TAM alternative polarization toward a protumor phenotype using macrophages isolated from tumor grafts. The protumor TAM phenotype, isolated from tumor grafts, was analyzed by flow cytometry staining with CD11b, F4/80 and CD206 and immunofluorescence. The intensity of CD206 staining was significantly higher in S100A4^WT^ TAMs compared with S100A4^KO^ TAMs derived from E7710 tumor grafts (figure 2I). Consistently, we found that CD206 expression levels in tumor tissues were also higher in E7710-bearing S100A4^WT^ mice compared with S100A4^KO^ mice (online supplemental figure 5A), suggesting that S100A4 sufficiency is mandatory for the expression of protumor markers in TAMs. Correspondingly, a reduction in the number of CD206^+^ protumor TAMs was also observed in S100A4-deficient TAMs by flow cytometry (online supplemental figure 5B). There are still F4/80/CD206 double-positive TAMs in germline S100A4 KO mice, indicating that KO S100A4 cannot completely block the TAMs’ M2-like polarization and that S100A4 is not the sole determinant during this process.

Next, we further evaluated the roles of macrophage-specific S100A4 on TAM polarization using E7710 tumor-bearing mice of the paired S100A4^M−WT^ and control S100A4^M−KO^ strains. Consistent with the observations in the germline S100A4 KO mice, flow cytometry analysis revealed that an S100A4-specific deficiency in macrophages also caused about a reduction in the frequencies of CD206^+^ macrophages (figure 2J). Compared with germline S100A4 KO, macrophage-specific S100A4 KO caused more decrease of the frequency of F4/80/CD206 double-positive TAMs. These in vivo data strengthened the conclusion that S100A4 is an essential factor in alternative polarization of TAMs toward a protumor phenotype.

### S100A4^+^ TAMs correlate with chemoresistance and poor prognosis of cancer patients

**Figure 3:**
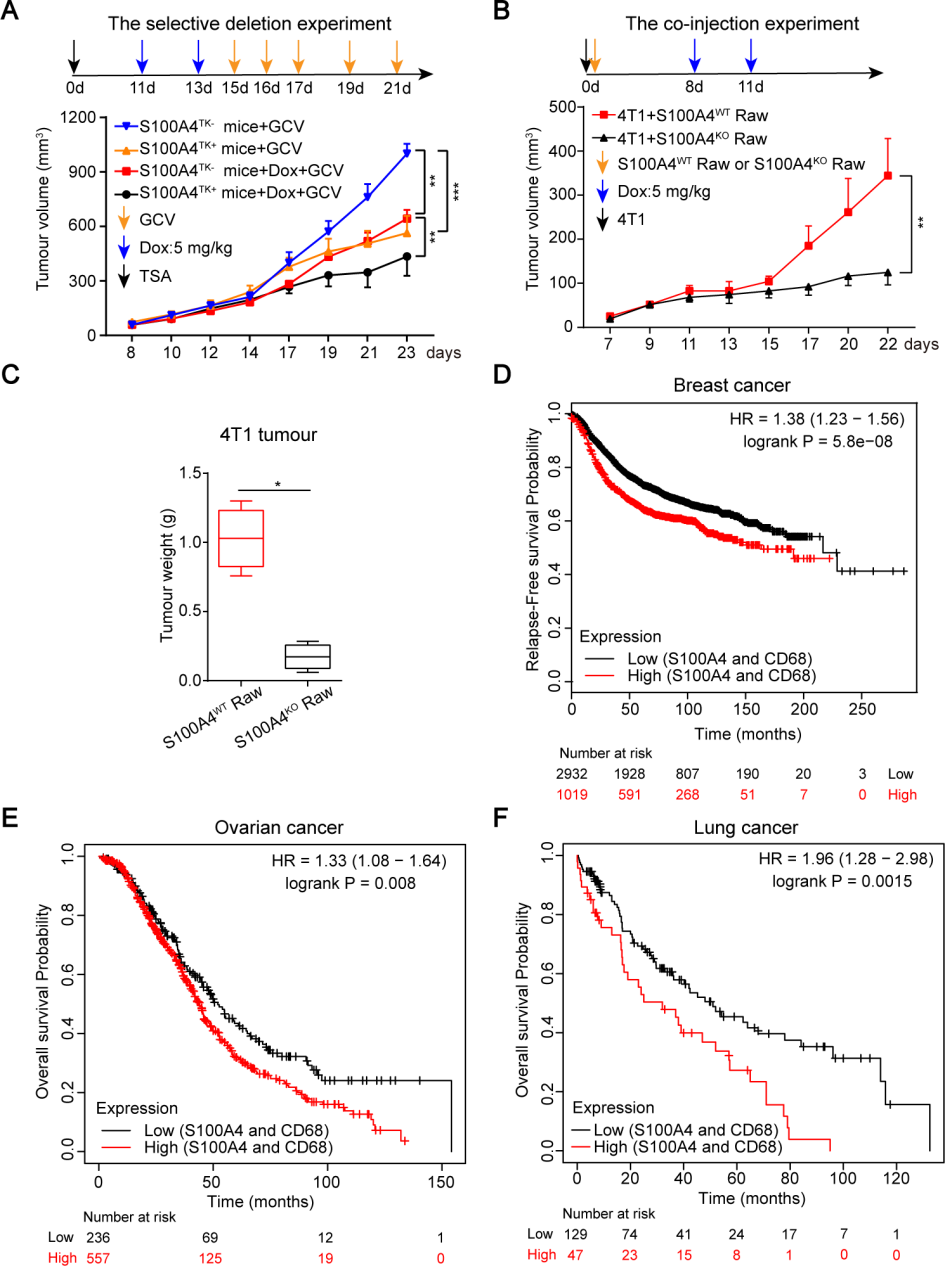
Macrophagic S100A4 correlates with chemotherapy-resistance and poor prognosis. (A) Effects of deleting proliferating S100A4^+^ cells on chemosensitivity. Growth of tumor grafts was monitored over time after initial injection of TSA breast cancer cells into S100A4^TK+^ transgenic mice and control S100A4^TK−^ littermates (n≥6). The mice were treated with doxorubicin (5 mg/kg) on days 11 and 13 before treatment with GCV (50 mg/kg body weight) on the indicated days. (B, C) Coinjection of 4T1 breast cancer cells together with S100A4^WT^ or S100A4^KO^ Raw264.7 cells (1:1 ratio) into WT Balb/c mice (n≥5) treated with doxorubicin (5 mg/kg) on days 8 and 11. Growth of tumor grafts was monitored over time after initial injection (B). Tumor weight of tumor grafts excised at the end of the experiment are shown (C). (D–F) Comparison of the relapse-free survival probability or overall survival rate of S100A4^low^/CD68^low^ and S100A4^high^/CD68^high^ patients with breast (D), ovarian (E), and lung (F) cancers after chemotherapy. Data are derived from a Kaplan-Meier plotter. Data (in A–C) are presented as mean±SE and were analyzed by unpaired Student’s t-test and two-way ANOVA Sidak's multiple comparisons. The data are from one representative experiment of three independent experiments (A–C). *P<0.05, **P<0.01, ***P<0.001. ANOVA, analysis of variance; GCV, ganciclovir; WT, wild type.

The presence of large numbers of TAMs correlates with poor response of tumors to anticancer agents and poor prognosis of cancer patients.^29 30^ Therefore, we wondered whether S100A4^+^ TAMs mediate therapeutic responses, thereby impacting prognosis and long-term patient survival. First, S100A4^TK+^ female mice, which express a truncated herpesvirus TK gene under the control of the *s100a4* promoter,^24^ and control S100A4^TK−^ littermates, were implanted with TSA breast cancers treated with or without doxorubicin (figure 3A). Without treatment, tumor growth was remarkably inhibited when proliferating S100A4^+^ cells were ablated by GCV treatment. After treatment, the relapse of tumor growth was identical in both mice strains before GCV treatment. In contrast, as expected, the abatement of proliferating S100A4^+^ cells by GCV treatment remarkably inhibited the regrowth of TSA tumor grafts (figure 3A). Thus, these results corroborated the previous conclusions that targeting S100A4 can improve the therapeutic efficacy of cancer treatment.

Based on the positive correlation between the frequencies of S100A4^+^ TAMs and tumor growth without chemotherapy (figure 2), next, we investigated the effects of S100A4^+^ TAMs on tumor growth after chemotherapy using paired S100A4^WT^ and S100A4^KO^ Raw264.7 cells. First, we tested the chemosensitivity of these cells to clinically used drugs doxorubicin and 5-fluorouracil. No difference in response to these two drugs was found between the two cell lines (online supplemental figure 6A, B). Then, the two cell lines together with 4T1 breast cancer cells were co-transplanted into WT Balb/c mice and treated with doxorubicin. After chemotherapy, compared with the coinjection of S100A4^KO^ Raw264.7 cells, the presence of S100A4^WT^ Raw264.7 cells markedly promoted 4T1 tumor regrowth and tumor weight increase (figure 3B, C). Thus, these results demonstrated that the presence of the S100A4^+^ TAM subset impedes anti-tumor response. Although S100A4 is a strong predictor of poor survival in cancer patients,^31–33^ whether S100A4^+^ TAMs are associated with prognosis and survival of patients with cancer remains unknown. To this end, we examined the Kaplan-Meier plotter dataset (https://kmplot.com/analysis/index.php?p=service). The database was established using available microarray gene expression data downloaded from Gene Expression Omnibus. The analysis results can be automatically generated based on the input information of genes or restrictions. The mRNA expression levels of S100A4 and CD68 were acquired according to the deposited microarray data in this database.^34^ We divided the patient cohort after therapy into two groups: S100A4^Low^/CD68^Low^ and S100A4^High^/CD68^High^, based on the expression levels of S100A4 and CD68, markers of TAMs. The Kaplan-Meier survival curve showed that greater expression of S100A4 and CD68 was predictive of reduced relapse-free survival of patients with breast cancer (figure 3D), and of reduced overall survival of patients with ovarian or lung cancer after chemotherapy (figure 3E, F). These results, therefore, indicate that S100A4 is a powerful predictor of cancer patient survival as a marker of protumor TAMs.

### S100A4 is needed for FAO upregulation during macrophage alternative polarization

To resolve the whole picture of S100A4 functions in alternative polarization of TAMs, we conducted quantitative proteomics analysis of IL-4-activated S100A4^WT^ and S100A4^KO^ BMDMs and identified ~200 differentially expressed peptides. Using the Kyoto Encyclopedia of Genes and Genomes (KEGG) and Gene Ontology (GO) analysis, we found that oxidative phosphorylation (OXPHOS) metabolism ranked at the statistically enriched pathways (false discovery rate (FDR)<0.5, (online supplemental figure 7A, B).

**Figure 4:**
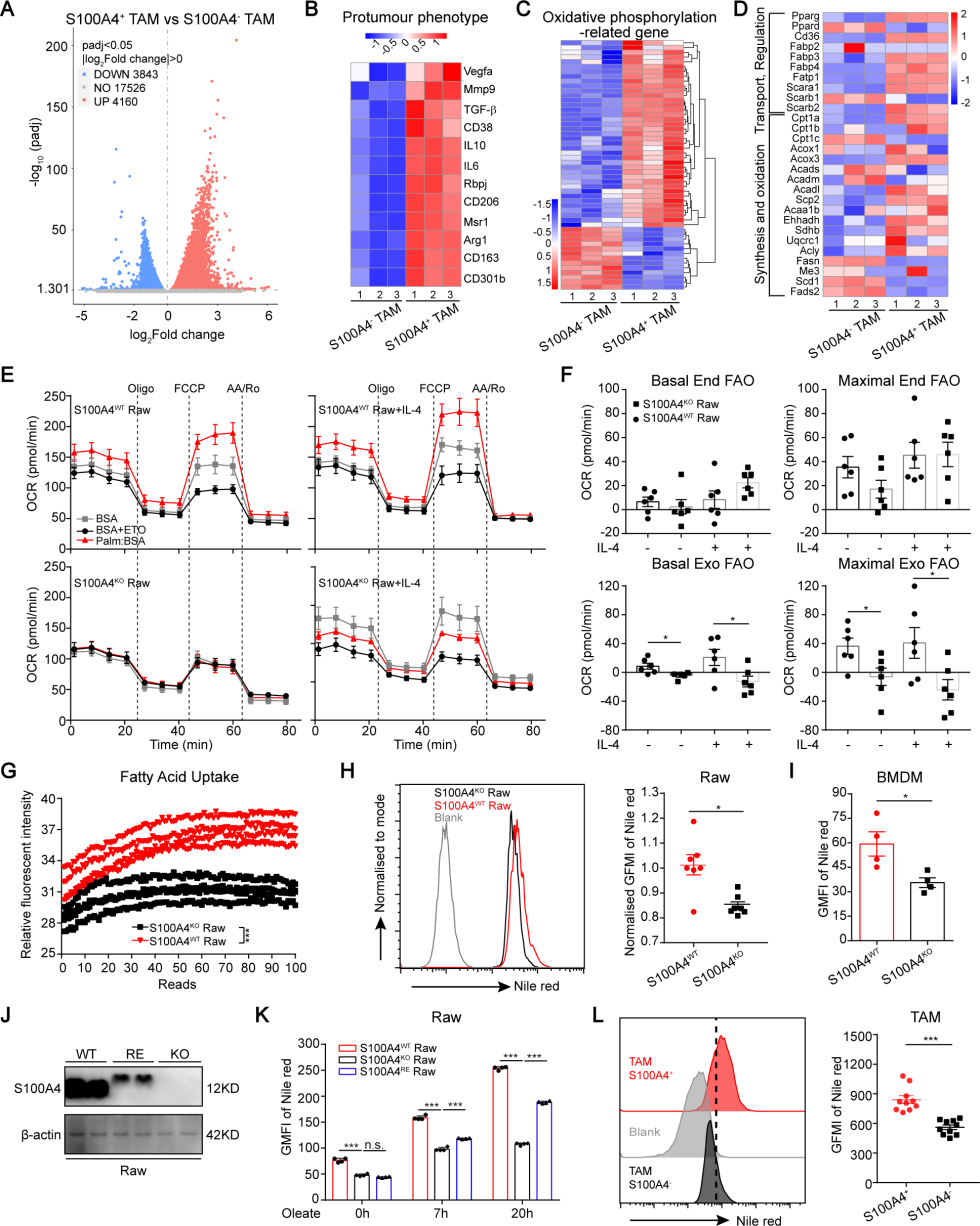
S100A4 depletion reduces macrophagic capability in usage of exogenous fatty acids. (A–D) RNA sequencing analysis of DEGs between S100A4^+^ and S100A4^−^ TAMs in tumor grafts from E0771 breast cancer cell-bearing S100A4^WT-EGFP^ reporter mice. Volcano diagram of DEGs, threshold is padj < 0.05 (A). Heatmap view of gene expression of M2 markers (B), oxidative phosphorylation (C) and FA metabolism (D) in S100A4^+^ and S100A4^−^ TAMs. (E, F) S100A4^WT^ or S100A4^KO^ Raw264.7 cells were activated by IL-4 for 36 hours. The non-activated macrophages were used as controls. The mitochondrial oxygen consumption rate (OCR) was monitored and analyzed in the presence or absence of FAO substrate (PALM, palmitate) or CPT1 inhibitor (ETO, etomoxir) via XFe^96^ Analyzer (E). The basal and the maximal endogenous or exogenous FAO was quantified based on the OCR value (F). (G) Measurement of FA uptake in S100A4^WT^ or S100A4^KO^ Raw264.7 cells. (H, I) Flow cytometric analysis of lipid content in S100A4^WT^ or S100A4^KO^ Raw264.7 cells (H) or in BMDMs (I) stained with Nile red. (J) Identification of the re-expression of S100A4 in S100A4^KO^ Raw264.7 cells. (K) Flow cytometric analysis of lipid content (Nile red staining) in S100A4^WT^, S100A4^KO^, or S100A4^RE^ Raw264.7 cells with oleate (0.2 mM) incubation. (L) Flow cytometric analysis of lipid content in S100A4^+^ or S100A4^−^ TAMs isolated from E0771 breast cancer cell-bearing female mice. Data are presented as mean±SE and were analyzed by two-way ANOVA with Tukey's multiple multiple comparisons and unpaired non-parametric Mann-Whitney U test. The data are from one representative experiment of three independent experiments (E–K). *P<0.05, ***P<0.001. ANOVA, analysis of variance; BMDMs, bone marrow-derived macrophages; DEGs, differentially expressed genes; FA, fatty acid; FAO, fatty acid oxidation; FCCP, carbonyl cyanide 4-(trifluoromethoxy) phenylhydrazone; GMFI, geometric mean fluorescence intensity; IL-4, interleukin 4; KO, knockout; Oligo, oligomycin; n.s., not signifacant; Rtn/AA, rotenone/antimycin-A; TAM, tumor-associated macrophage; TGF-β, transforming growth factor-β; WT, wild type.

To further compare detailed changes to the gene expression signature, we performed protein-coding mRNA-seq analysis of S100A4^+^ and S100A4^−^ TAMs isolated from tumor grafts of E7710 tumor-bearing S100A4^WT−EGFP^ reporter mice (online supplemental figure 8A). A total of 8003 differentially expressed genes (DEGs) were identified, including 4160 up-regulated and 3843 downregulated genes (figure 4A; padj <0.05). KEGG and GO analysis of the DEGs from RNA-seq data further revealed that the molecular signatures of S100A4^+^ TAMs were identical to the ones observed in S100A4^WT^ Raw264.7 cells or BMDMs (figure 4B). Consistent with the proteomics analysis, gene expression in OXPHOS metabolism was also statistically changed in RNA-seq between S100A4^+^ and S100A4^−^ TAMs (figure 4C). Interestingly, S100A4^+^ TAMs had higher expression of genes related with FA metabolism pathway, especially FA transport, compared with S100A4^−^ TAMs. The expression of genes related with FAO was not significantly changed between S100A4^+^ and S100A4^−^ TAMs (figure 4D), which was further confirmed in S100A4^WT^ and S100A4^KO^ BMDMs (online supplemental figure 8B). In contrast, compared with S100A4^−^ TAMs, S100A4^+^ subsets had significantly downregulated genes in FA synthesis (figure 4D).

Our previous work revealed that intracellular lipid droplets, which provide a stable source of FAs for TAMs, control TAMs polarization toward an M2-like phenotype.^11^ Another research group also found that enhanced lipid accumulation and metabolism are required for the differentiation and activation of TAMs.^10^ Considering that FA catabolism involves extra-FA transport into cells and mitochondrion-dependent FAO, we wondered which of these processes is affected by S100A4 deficiency. Accordingly, we compared mitochondrial FAO capacity by measuring the (OCR, index of OXPHOS) in S100A4^WT^ and S100A4^KO^ Raw264.7 cells with or without the addition of BSA-palmitate (PALM, the FAO substrate) or BSA-ETO (the FAO inhibitor figure 4E). We observed that the basal and maximal OCR of S100A4^WT^ Raw264.7 cells, treated with or without IL-4, in media without FAO substrate was not significantly different from those of S100A4^KO^ Raw264.7 cells, indicating S100A4-KO did not affect the endogenous FA oxidation (figure 4F, upper row). Intriguingly, however, the respiratory capacity of S100A4^KO^ Raw264.7 cells with or without IL-4 treatment was greatly impaired when the FAO substrate was present (figure 4F, lower row), manifesting that S100A4-KO only impaired the exogenous FA oxidation. However, what we did not observe was a significant decrease of expression in FAO-related genes in S100A4-deficient BMDMs (online supplemental figure 8B). Taking these findings together, we concluded that S100A4 deficiency results in major defects in extracellular FA usage, causing FA shortage during FAO of macrophage alternative polarization.

Next, we explored whether the failure of FAO upregulation in IL-4-activated S100A4^KO^ Raw264.7 cells was due to defects in FA transportation pathways. Examination of the FA uptake discovered that S100A4 deficiency in Raw264.7 cells led to a significant reduction in FA absorption (figure 4G). To further confirm the above findings, we added oleate in cell culture media to observe intracellular accumulation of lipid droplet (LD), because excessively absorbed FAs can be stored within cells as LDs. Consistently, flow cytometry analysis revealed that a close correlation between S100A4 deficiency and decreased intracellular lipid content was also observed in S100A4-deficient Raw264.7 cells and BMDMs (figure 4H, I), indicating that S100A4 is necessary for FA uptake by macrophages.

To further explore whether the re-expression of S100A4 can restore FA absorption, we re-constituted S100A4 expression in S100A4^KO^ Raw264.7 cells by transfecting S100A4 into this cell line. Flow cytometric analysis indicated that the lipid contents in S100A4^RE^ Raw264.7 cells with S100A4 re-expression were significantly restored (figure 4J, K). A reduction of lipid content was also observed in S100A4-deficient TAMs derived from E0771 tumor grafts (figure 4L). Taken together, these data suggest that S100A4 is required for the reprogramming of FA metabolism in macrophages, which governs macrophage polarization.

### S100A4 controls TAM alternative polarization via determining PPAR-γ induction

It is well known that the transcriptional factors of STAT6,^35 36^ PPAR-γ^9 13^ and PPAR-γ-coactivator-1β (PGC-1β)^37^ control FAO upregulation and mitochondrial biogenesis in macrophage alternative polarization. Therefore, we first examined protein levels of total and phosphor-STAT6 in S100A4^WT^ and S100A4^KO^ Raw264.7 cells with or without IL-4 treatment. No differences in expression and activation of STAT6 were found between the two cell lines (online supplemental figure 9A). However, the IL-4-induced S100A4 upregulation was significantly inhibited in the presence of STAT6 inhibitor (online supplemental figure 9B). In contrast, RNA-seq analysis revealed that there was a significantly decreased expression of PPAR-γ, but not of PPAR-α and PPAR-β/δ, in S100A4^−^ TAMs (CD11b and F4/80 double positive cells) compared with S100A4^+^ TAMs (online supplemental figure 9C). These results indicated that S100A4/PPAR-γ is downstream pathway and regulated by STAT6 signaling.

**Figure 5:**
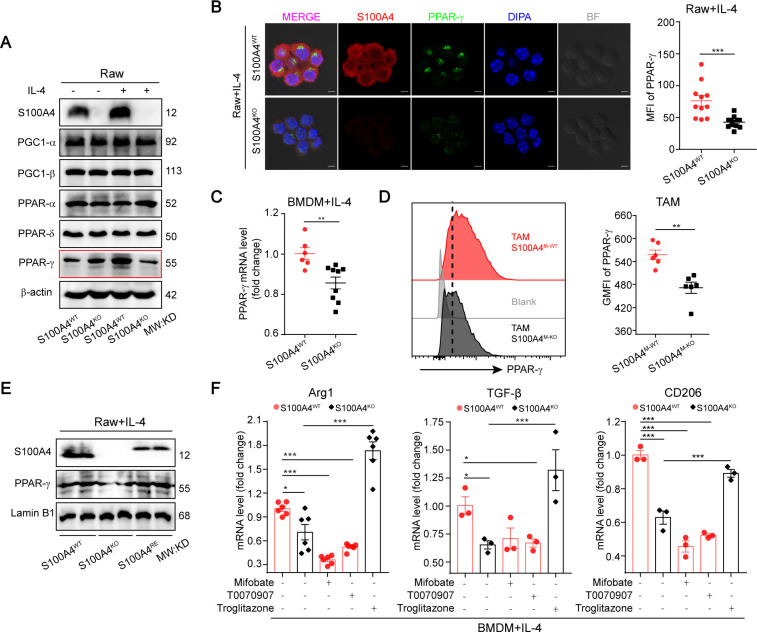
S100A4 controls macrophage alternative activation via determining PPAR-γ induction. (A) Immunoblotting examination of expression of PPARs and their co-activators in S100A4^WT^ or S100A4^KO^ Raw264.7 cells treated with or without IL-4. (B) Immunofluorescence analysis of expression of S100A4 and PPAR-γ in IL-4-activated S100A4^WT^ or S100A4^KO^ Raw264.7 cells. The immune complexes were detected with a secondary antibody conjugated to Alexa Fluor 555 (red). DNA was stained with DAPI (blue). Scale bars, 5 μm. (C) q-PCR analysis of PPAR-γ expression in IL-4-activated S100A4^WT^ or S100A4^KO^ BMDMs. (D) Flow cytometric analysis of PPAR-γ expression in TAMs of the tumor grafts isolated from E0771 breast cancer cell-bearing S100A4^M-WT^ or S100A4^M-KO^ mice. (E) Immunoblotting examination of PPAR-γ expression in IL-4-activated S100A4^WT^, S100A4^KO^ or S100A4^RE^ Raw264.7 cells. (F) q-PCR analysis of M2 marker expression in IL-4-activated BMDMs in the presence or absence of PPAR-γ inhibitor (mifobate, 200 µM; or T0070907, 100 µM) or agonist (troglitazone, 2 µM). Data are presented as mean±SE and were analyzed by one-way ANOVA with Tukey's multiple comparisons. The data are from one representative experiment of three independent experiments (A, B, E). *P<0.05, **P<0.01, ***P<0.001. ANOVA, analysis of variance; BMDMs, bone marrow-derived macrophages; GMFI, geometric mean fluorescence intensity; KO, knockout; IL-4, interleukin 4; MFI, mean fluorescence intensity; PPAR-γ, peroxisome proliferator-activated receptor γ; TAM, tumor-associated macrophage; WT, wild type; DAPI, 4',6-diamidino-2-phenylindole.

Such expression patterns of PPARs were further confirmed in S100A4^WT^ and S100A4^KO^ Raw264.7 cells by immunoblotting, in which the IL-4−stimulated induction but not the expression of PPAR-γ was inhibited by S100A4 deficiency. In contrast, the expression and induction of other PPARs (PPAR-α and PPAR-β/δ) and PGC-1s (PGC-1α and PGC-1β) was not affected by S100A4 deficiency (figure 5A). The S100A4-dependent PPAR-γ induction in macrophage alternative polarization was further confirmed in S100A4-deficient Raw264.7 cells, BMDMs and macrophage specific S100A4-KO TAMs (figure 5B–D). When we reconstituted S100A4 expression in S100A4-deficient Raw264.7 cells, IL-4−stimulated PPAR-γ induction was restored to the same levels as in S100A4^WT^ Raw264.7 cells (figure 5E). Therefore, these results clearly demonstrated that S100A4 is required―that is, it is both necessary and sufficient―for PPAR-γ induction responding to M2 or M2-like macrophage activators.

Next, we investigated whether PPAR-γ exerted the same roles in the S100A4^+^ subsets of TAMs as in other TAMs, as reported previously. We inhibited PPAR-γ activation in S100A4^WT^ BMDMs with a selective PPAR-γ inhibitor (mifobate or T0070907); simultaneously activated PPAR-γ in S100A4^KO^ BMDMs with PPAR-γ agonist (troglitazone) in combination with IL-4. The PPAR-γ inhibitors dramatically blocked the upregulation of M2 marker (arginase, TGF-β, and CD206) expression in S100A4^WT^ BMDMs. In contrast, the PPAR-γ agonist reversed the expression of M2 marker genes (figure 5F). The highly positive correlation between S100A4 expression and PPAR-γ activation indicated that the PPAR-γ pathway is involved in the protumor polarization of S100A4^+^ subsets.

### S100A4-PPAR-γ facilitates FA uptake of TAMs through CD36

**Figure 6:**
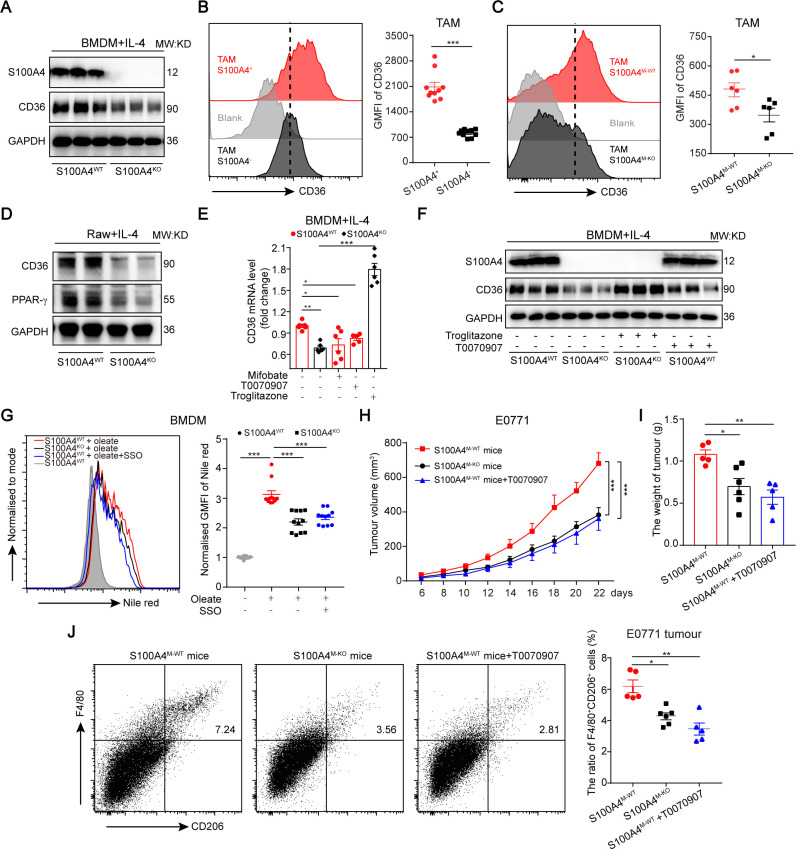
S100A4 promotes fatty acid uptake through upregulation of PPAR-γ targeting gene CD36. (A) Immunoblotting examination of CD36 expression in IL-4−activated S100A4^WT^ or S100A4^KO^ BMDMs. (B) Flow cytometric analysis of CD36 expression in S100A4^+^ and S100A4^−^ TAMs from tumor grafts in E0771 breast cancer cell-bearing S100A4^WT−EGFP^ reporter mice. (C) Flow cytometric analysis of CD36 expression in S100A4^M−WT^ and S100A4 ^M−KO^ TAMs from tumor grafts in E0771 breast cancer cell-bearing S100A4^M−WT^ or S100A4^M−KO^ female mice (n≥5). (D) Immunoblotting examination of CD36 expression in IL-4-activated Raw264.7 cells. (E, F) Examination of CD36 mRNA and protein expression in IL-4-activated S100A4^WT^ or S100A4^KO^ BMDMs in the presence or absence of PPAR-γ inhibitor (mifobate, 200 µM; or T0070907, 100 µM) or agonist (troglitazone, 2 µM). (G) Flow cytometric analysis of lipid content in S100A4^WT^ or S100A4^KO^ BMDMs stained with Nile red. The cells were incubated with oleate (0.2 mM) in the presence or absence of CD36 inhibitor SSO (25 µM) as indicated. Data are from three independent experiments. The values were normalized with that in S100A4^WT^ BMDMs without treatment. (H, I) E0771 breast cancer cells were implanted into S100A4^M−WT^ (with or without PPAR-γ inhibitor, T0070907) or S100A4^M−KO^ female mice. The growth of tumor grafts (H, left) was monitored over time after initial cell implantation, and the tumor weight (I, right) of tumor grafts excised at the end of the experiment are shown. The number of CD206^+^ protumor TAMs (J) in tumor grafts was examined by flow cytometry. Data are presented as mean±SE and were analyzed by unpaired nonparametric Mann-Whitney U test in B, C; one-way ANOVA with Tukey's multiple comparisons test in E, G–J; and two-way ANOVA with Dunnett's multiple comparisons test in H. The data are from one representative experiment of three independent experiments (A–D, F, J). *P<0.05, **P<0.01, ***P<0.001. ANOVA, analysis of variance; BMDMs, bone marrow-derived macrophages; GMFI, geometric mean fluorescence intensity; KO, knockout; IL-4, interleukin 4; MFI, mean fluorescence intensity; PPAR-γ, peroxisome proliferator-activated receptor γ; TAM, tumor-associated macrophage; WT, wild type; SSO, sulfo-N-succinimidyl oleate Na.

Many target genes of PPAR-γ are effectors of macrophage alternative polarization.^38^ To determine which of these effectors specifically act downstream from S100A4-PPAR-γ during macrophage alternative polarization, using IL-4−activated S100A4^WT^ and S100A4^KO^ BMDMs we re-checked the PPAR-γ−regulated DEGs in RNA-seq data by q-PCR. We found that only CD36 expression was markedly inhibited by S100A4 deficiency (online supplemental figure 10A). This was further verified by immunoblotting at the protein level (figure 6A). A correlation between S100A4 deficiency and CD36 downregulation was also found in S100A4^−^ and S100A4^+^ TAMs isolated from E0771-bearing S100A4^WT−EGFP^ reporter mice (figure 6B), and in macrophage-specific S100A4-KO TAMs (figure 6C). Using S100A4^WT^ and S100A4^KO^ Raw264.7 cells, we observed that CD36 induction was impaired in KO cells (figure 6D and online supplemental figure 10B). Under abdominal inflammation conditions, the proportion of CD36^+^ macrophages isolated from the spleen of S100A4^KO^ mice was also significantly decreased compared with that derived from S100A4^WT^ mice (online supplemental figure 10C). These data demonstrated that macrophagic S100A4 is also necessary for CD36 induction.

Using IL-4-activated S100A4^WT^ and S100A4^KO^ BMDMs, we found that PPAR-γ inhibition could inhibit CD36 expression in S100A4^WT^ BMDMs. In contrast, the PPAR-γ agonist reversed the expression of CD36 in S100A4^KO^ BMDMs to similar levels as in S100A4^WT^ BMDMs (figure 6E, F and online supplemental figure 11). Flow cytometry analysis of lipid levels in S100A4^M−WT^ or S100A4^M−KO^ BMDMs revealed that CD36 inhibition could indeed significantly impair lipid accumulation in S100A4-sufficient cells (figure 6G). These data indicated that CD36 is the major effector that contributes to S100A4-mediated FA uptake.

We further compared the effects of depletion of S100A4^+^ TAMs and inhibition of PPAR-γ on tumor growth. To this end, we implanted E0771 and MCA205 tumor cells into S100A4^M−KO^ and control S100A4^M−WT^ mice with or without PPAR-γ inhibitor. Significantly inhibited tumor development (tumor growth and weight) and a lower proportion of CD206^+^ protumor TAMs was observed in the macrophagic-specific S100A4-KO animals and in the animals treated with PPAR-γ inhibitor compared with control animals (figure 6H, I, J and online supplemental figure 12A, B), in which no body weight change was observed (online supplemental figure 12C, D). These data clearly indicated that S100A4-PPAR-γ upregulation of TAM FAO is through CD36, suggesting that depletion of the S100A4^+^ subset population of TAMs has highly promising therapeutic potential in cancer therapy.

## Discussion

A major challenge in understanding how the TME shapes TAMs with protumor functions is deciphering the mechanisms underlying TAM alternative activation towards a protumor phenotype, which links macrophage plasticity and function.^39^ This study provides evidence revealing how S100A4 controls FA catabolism reprogramming to modulate macrophage protumor polarization. Due to the requirement of PPAR-γ induction in this process,^13^ this study thus suggests that blocking macrophagic S100A4 may reverse this alternative polarization toward a protumor phenotype and thereby reduce the effects of TAMs on tumor initiation, malignancy and cancer treatment resistance.

PPARs serve as metabolic sensors to regulate lipid homeostasis on activation by a diverse spectrum of FAs and FA derivatives, such as palmitic acid.^38^ PPAR responsive elements exist abundantly in genes induced in M2 macrophages.^40^ Although in metabolic disorder conditions the polarization of adipose and liver-resident macrophages toward the M2 phenotype is dependent on PPAR-β/δ,^41 42^ in a tumor context PPAR-γ is not only necessary but also sufficient for TAM polarization towards a protumor phenotype.^13 43^ Our findings demonstrated that S100A4 deficiency solely prevented induction of PPAR-γ, not of other PPARs and PGCs, reflecting that S100A4 plays a parallel role in macrophage protumor polarization to PPAR-γ.

Our previous work identified that macrophage polarization toward a protumor phenotype is dependent on accumulation of intracellular lipid droplets, which provide a stable source of FAs for TAMs.^11^ The source of the FAs that support such lipid droplet accumulation and metabolic programming appears to be taken up via CD36.^43^ CD36 fuelling of FAO has an important role in TAM polarization with protumor characteristics.^43^ Remarkably, CD36 amplification positively correlates with metastasis in a large amount of human cancers, such as melanoma.^44^ Our finding that CD36 is the major effector of the S100A4/PPAR-γ pathway reinforces the important role of CD36-mediated FA uptake in macrophage protumor polarization. Increased FA levels are found in various TME such as breast cancers, thus our findings suggest that blocking S100A4/PPAR-γ pathway in such cancer types with FA-enriched TME may have better effects than in cancers with nutrient-stressed TME.

Our RNA-seq analysis revealed that S100A4^+^ TAMs exhibited a typical M2-like gene expression signature. Therefore, it is logical that any environmental cues that can induce S100A4 upregulation may induce TAM protumor polarization. Until now, a plethora of TME components―including TGF-β, Wnt and hypoxia inducible factor-1α (HIF-1α)―have shown potent effects on S100A4 induction.^26–28^ Therefore, the more inducible factors of S100A4 that are discovered, the better the mechanistic basis of M2 polarization may be understood in the future.

The M2-like TAM facilitate further tumor growth and malignancy can be ascribed to their ability to suppress T cell responses within TME.^4^ Protumor TAMs can directly regulate T cell function by multiple mechanisms, including checkpoint engagement via their expression of immune checkpoint molecules such as programmed cell death-ligand 1 (PD-L1), suppression of CD8^+^ T cell function by production of inhibitory cytokines (such as IL-10 and TGF-β), and impairment of T cell proliferation by depleting L-arginine by their high levels of Arginase 1.^45^ S100A4^+^ TAMs express high levels of PD-L1, arginase 1 and TGF-β, reflecting that they have immune-suppressive capability.

Resistance to chemotherapy is not only ascribed to intrinsic features of tumor cells, but also conferred by non-malignant stromal cells in TME, especially TAMs.^46^ M2-like TAMs in solid tumors are associated with poor prognosis and correlates with chemotherapy resistance in most cancers.^47^ It has been reported that overexpression of S100A4 in malignant cancer cells is associated with chemoresistance.^48 49^ This study implied that overexpression of S100A4 in non-malignant stromal cells also induce chemotherapy resistance through driving polarization of S100A4^+^ TAMs toward M2-like protumoral phenotypes.

The M2-like protumor phenotype of TAMs is reversible in most observed cancers.^50^ Metabolic reprogramming holds potential for modulating macrophage phenotypes and developing new therapeutic approaches.^50^ Our current analysis of clinical human cancer data showed greater expression of S100A4 and CD68 to be a strong predictor of poor prognosis for cancer patients. Therefore, the role of S100A4-dependent PPAR-γ induction as a mechanism to reduce tumor immunogenicity and evade immune surveillance deserves further exploration.

10.1136/jitc-2021-002548.supp2 Supplementary data

## Data Availability

The RNA-sequencing data have been deposited into the GEO repository with the GEO Submission code GSE161060.
