# Supplementary material for: S100A4 enhances protumor macrophage polarization by control of PPAR-γ-dependent induction of fatty acid oxidation
Source: J Immunother Cancer. 2021 Jun 18;9(6):e002548. doi: 10.1136/jitc-2021-002548 (PMC8215236; doi:10.1136/jitc-2021-002548)
Supplement: Supplementary data [file jitc-2021-002548supp001.pdf]

Supplementary Materials

Supplementary materials and methods

Murine tumor models

Adherent breast cancer cells (TSA, E7710, and 4T1) were harvested and washed twice with phosphate-buffered saline (PBS). Single-cell suspensions of 5×10^5 cells were injected into the mammary pads of 6–8-week-old female mice with various genetic backgrounds. MCA205 fibrosarcoma cells (5×10^5/per mouse) were implanted into the back muscles of 6–8-week-old male mice. For co-injection chemotherapy model, 4T1 breast cancer cells (5×10^5) together with S100A4^{WT} or S100A4^{KO} Raw264.7 cells (5×10^5) were co-injected at a ratio of 1:1. For adoptive transferal model, 2×10^6 S100A4^{WT} or S100A4^{KO} Raw264.7 cells were injected into the caudal veins of mice.

Tumour volumes were measured every 2 days with a caliper and calculated using the equation: $V = 0.5 \times (\text{minor tumour axis})^2 \times (\text{major tumour axis})$. For flow cytometric analysis or cell sorting specific subset population of infiltrated immune cells in tumour, mice were sacrificed after 12–17–day of the tumour cell inoculation, tumours were weighed, and the infiltrated immune cells were collected. For S100A4^{TK+} and S100A4^{TK-} murine model, the intraperitoneal injection of GCV (50 mg/kg) were given for 3 days continuously, and then once every 2 days till the end of experiment. For intraperitoneal injection of PPAR-γ inhibitor, after 8-day of the tumour-implantation, the tested mice were given T0070907 (2 mg/kg) once a day for 2 weeks.

Flow cytometry analysis

Liu S, *et al. J Immunother Cancer* 2021; 9:e002548. doi: 10.1136/jitc-2021-002548

Macrophagic S100A4 enhances protumour macrophage polarization

Single-cell suspensions were prepared from tumour tissues, Raw264.7 cells, or BMDMs. The collected cells were blocked with 3% BSA in PBS for 30 minutes. For staining cell surface proteins, the cells were incubated with the corresponding flow cytometric antibodies or isotype IgG control antibody for 30 minutes at 4 °C. For staining intercellular proteins, the cells need to be fixed with intracellular fixation & permeabilization buffer (eBioscience, San Diego, California, USA) for 30 minutes, washed with permeabilization buffer (Invitrogen, Carlsbad, California, USA), and collected at 500 *g* for 5 minutes at 4 °C. Cells were then resuspended and incubated on ice with the corresponding flow cytometric antibodies or isotype IgG control antibody for 30 minutes at 4 °C, followed by washing with permeabilization buffer, and collected at 3000 rpm for 5 minutes at 4 °C. Then, the cells were analyzed by using a FACSCalibur or FACSAria IIIu (BD Biosciences, San Jose, California, USA) flow cytometer. The flow cytometry data were analyzed using FlowJo V10 software (TreeStar, Ashland, Oregon, USA).

Measurement of exogenous and endogenous fatty acid oxidation (FAO)

The respiration changes, caused by utilization of exogenous FAs, endogenous FAs, or uncoupling by FAs, were simultaneously measured using kit that works with the Seahorse XF analyzer and can simultaneously measure oxidation of exogenous and endogenous fatty acids (Agilent Technologies). The tested S100A4^{WT} and S100A4^{KO} Raw264.7 cells (10,000/well) were cultured on the XF^{96} cell culture microplate (102601-100, Agilent, Palo Alto, California, USA) and were stimulated with or without IL-4 (20 ng/mL) for 36 hours. Then the cell culture medium was changed to substrate-limited medium and cells were further cultured in this medium for 7 hours. The cells were washed with FAO assay medium once and incubated for 30 minutes in 37°C cell incubator without CO_2. The following test steps were according to

[illegible]

manufacturer's protocol. Oxygen consumption rate (OCR) was automatically calculated by the Seahorse XF-96 software in response to 2.5 μg/mL oligomycin, 0.8 μM FCCP, 2 μM rotenone plus 4 μM antimycin A, and 40 μM etomoxir (ETO, Sigma-Aldrich, Darmstadt, Germany).

Antibodies

CD45-Percp-Cy5.5 (#550994), CD11b-APC-Cy7 (#557657), CD11b-Percp-Cy5.5 (#550993), NK1.1-APC-Cy7 (#560618), CD8-Pacific Blue (#558106), Gr1-PE-Cy7 (#552985) and MHC-II(I-Ab)-PE (#553552) were from BD Pharmingen (San Diego, California, USA). F4/80-PE-Cy7 (#123114), F4/80-APC (#123116), B220-APC (#103212), CD4-PE (#100408), Gr1-PE (#108408), CD36-PE (#102605), CD36-Percp-Cy5.5 (#102619), CD206 (#141701), CD206-PE (#141705) and CD206-APC (#141707) were from Biolegend (San Diego, California, USA). S100A4 (ab41532), LaminB1 (ab16048), PGC1-α (ab54481), PGC1-β (ab176328), PPAR-α (ab24509), PPAR-δ (ab23673) and PPAR-γ (ab45036) were from Abcam (Cambridge, UK). STAT-6 (#9362) and P-STAT6 (#9361) were from Cell Signaling Technology (Danvers, Massachusetts, USA). β-actin (KM9001) and HRP-conjugated goat anti-mouse IgG (LK2003) were from Sungene Biotech (Tianjin, China). HRP-conjugated GAPDH (AC035) and HRP-conjugated goat anti-Rabbit IgG (AS014) were from Life Technologies (Carlsbad, California, USA). The Alexa Fluor 555 donkey anti-mouse IgG (A31572) and Alexa Fluor 555 donkey anti-rabbit IgG (A31570) were from Invitrogen (Carlsbad, California, USA). Mouse PD-L1-PE (12-5982-82) was from eBioscience (San Diego, California, USA). Mouse CD31 (NB100-2284) was from Novus Biologicals (Building IV Centennial, Colorado, USA).

Reagents

All cell culture media were from HyClone (Logan, Utah, USA) and all transfection reagents and Nile red (N1142) were from Invitrogen (Carlsbad, California, USA). Chemicals, including lipopolysaccharide (LPS, L4516), oleate (07501-5G), sulfo-N-succinimidyl oleate Na (SML2148) and 5-fluorouracil (5-Flu, F6627-1G) were from Sigma (Darmstadt, Germany).

Recombinant mouse S100A4 protein (4138-S4-050) and IL-4 (404-ML-010) were from R&D Systems™ (Minneapolis, Minnesota, USA). Recombinant murine M-CSF (315-02) and IFN-γ (315-05) were from Peprotech (Cranbury, New Jersey, USA). T0070907 (HY-13202), mifobate (HY-100277) and troglitazone (HY-50935) were from Med Chem Express (Monmouth Junction, New Jersey, USA). Trlzol reagent (15596026) was from Thermo Fisher Scientific (Waltham, Massachusetts, USA). Collagenase NB4 Standard Grade (17454) was from SERVA Electrophoresis (Heidelberg, Germany). 4′,6-diamidino-2-phenylindole (DAPI) (C0065) was from Solarbio (Beijing, China). Doxorubicin (Dox, A603456-0025) was from Sangon Biotech (Shanghai, China). Phenylmethanesulfonyl fluoride (PMSF) solution (B111-01) was from GeneStar (Beijing, China). Protease inhibitor cocktail (04693132001) and phosphatase inhibitor cocktail (04906837001) were from Roche (Basel, Switzerland). Cell counting kit-8 (CCK-8, ab228554), free fatty acid uptake assay kit (ab176768) and arginase activity assay kit (ab180877) were from Abcam (Cambridge, UK). GCV was from HuBeiKeYi Pharmaceutic Corporation (Hubei, China).

Cell lines with culture conditions and transfection

All of the cell lines were maintained in Dulbecco's modified Eagle's Medium (DMEM) supplemented with 10% fetal bovine serum (FBS), authenticated by single nucleotide polymorphism testing and mycoplasma contamination testing. Human 293FT, murine Raw264.7, and 4T1 breast cancer cells were from ATCC (Manassas, Virginia, USA). TSA breast cancer cells are derived from BALB/c background. Murine MCA205 fibrosarcoma and E0771 breast cancer cells are derived from C57BL/6 background. S100A4 knockout (S100A4^{KO}) Raw264.7 cell line was generated using single guide CRISPR–Cas9 (Cas9/sgRNA)-mediated deletions. Two sgRNAs targeting sequences were selected as follows: S100A4-sgRNA-1-Forward, 5′-ATT GCA CAT CAT GGC AAT GC-3′; S100A4-sgRNA-1-Reward, 5′-GCA TTG CCA TGA TGT GCA AT-3′; S100A4-sgRNA-2-Forward, 5′-AGC TCA AGG AGC TAC TGA CC-3′; and S100A4-sgRNA-2- Reward, 5′-GG TCA GTA GCT CCT TGA GCT-3′. The sgRNA cloning vector is pSpCas9 (BB)-2A-GFP (PX458) (Addgene

#48138). S100A4 re-expressing (S100A4^{RE}) Raw264.7 cells were generated by transfection with S100A4-expressing plasmids in a pCDH-EF1-MCS-T2A-copGFP lentiviral vector, which were constructed using the synthesized full-length S100A4 cDNA. This vector was packaged together with pMD2.G or psPAX2 vectors in human 293FT cells by lentiviral transduction particles.

Real-time quantitative PCR (q-PCR)

Total RNA was harvested from mouse tissues with Trizol reagent (Invitrogen). First-strand cDNA synthesis was performed using One-step RT-PCR kit (A215-01, GenStar, Beijing, China)). Then cDNA was used as a template for q-PCR reactions using Taqman primer-probes against specified mRNA transcripts (Applied Biosystems, Waltham, Massachusetts, USA). Reactions were performed using 2 × Real Star Green Power Mixture (A311-10, GenStar, Beijing, China). FAM channel intensity was normalized to ROX intensity, and C_t values were calculated using automatically determined threshold values using SDS software (Applied Biosystems). The relative expression of genes was calculated by a standard curve method and normalized to the expression level of GAPDH. Gene-specific PCR primers are listed in online supplemental Table 1.

Protein extraction and western blotting

For western blotting, cells were placed on iced and washed twice with chilled PBS. Proteins were extracted with RIPA buffer (Cell Signaling Technology) plus protease inhibitor cocktail and phosphatase inhibitor cocktail (Thermo Fisher Scientific). The protein concentration was determined with the BCA protein assay kit (Thermo Fisher Scientific). An estimated 15-50 μg proteins were loaded per well on SDS-PAGE gel and transferred onto PVDF membrane (Thermo Fisher Scientific). Membranes were washed with 1 × TBST, blocked with 5% BSA in TBST at room temperature for 1 hour, and incubated in first antibody as indicated in each figure and its legend. Next day, the membranes were washed 3 times with 1 × TBST, incubated with secondary antibody at 1:5000 dilution in 1 × TBST for 2 hours, and washed 3

times with 1 × TBST. Protein was visualized with enhanced chemiluminescence (Thermo Fisher Scientific). Densitometry was calculated using Image J software (Media Cybernetics, Bethesda, MD, USA).

Immunofluorescence staining and immunohistochemistry

Cells were washed and fixed with 4% paraformaldehyde in PBS for 15 minutes, permeabilized with PBS/0.1% Triton X-100 for 10 minutes, and blocked with 3% BSA in PBS for 1 hour. The permeabilized cells were then incubated overnight at 4°C with the primary antibody against S100A4 (1:200) or PPAR-γ (1:200) in PBS containing 3% BSA. Cells were washed three times with PBS/0.1% Tween 20, and then incubated for 1 hour with a fluorescence-conjugated secondary antibody at a dilution of 1:300 at 37°C. After 45 minutes, the cells were washed with PBS/0.1% Tween 20, and counterstained with DAPI to detect DNA. A confocal laser scanning microscope (LSM700, ZEISS, Jena, Germany) was used.

For immunohistochemistry, the murine tissues were fixed in 4% paraformaldehyde (pH 7.4) overnight, embedded in paraffin, and serially sectioned at 5 μm. For immunofluorescent staining, the sections were then incubated with primary antibodies against CD206 (1:200) and CD31 (1:200) in PBS with 3% BSA overnight at 4°C. The tissue sections were washed three times with PBS and then incubated for 1 hour with fluorescence-conjugated Alexa Fluor 555 goat anti-rat or Alexa Fluor 555 donkey anti-rabbit secondary antibody at a dilution of 1:300 at 37°C. Images of the stained tissue samples were obtained using an OLYMPUS DP71 microscope (Tokyo, Japan). The fluorescence intensity was calculated by the Image J software.

Macrophage preparation and activation

BMDMs were prepared from BM cells, isolated from mouse femur. Then the BM cells were cultured in 10 cm dishes with DMEM containing 20% FBS and M-CSF (50 ng/mL) medium for 6–7 days. The adherent macrophages were harvested and cultured for further experiments. Positive selection of macrophage was conducted by the flow-cytometric analysis. For the activation experiments, macrophages were stimulated with IL-4 (20 ng/mL) for 36

hours, or with LPS (100 ng/mL) in combination with IFN-γ (20 ng/mL) for 12 hours.

Arginase activity assay

Relative arginine levels were measured using a colorimetric arginase activity assay kit according to manufacturer's instructions (ab180877, Abcam). The isolated cells were washed with PBS and were resuspended in assay buffer with 0.01% of Triton X-100 to disrupt the cell membrane. The supernatant samples were diluted 1:10 and 40 μL incubated with the arginase enzyme. After 10 minutes, the absorbance was measured at 570 nm in kinetic mode in an Epoch 2 microplate reader (BioTek, Winooski, Vermont, USA). The values were compared with an arginine standard curve.

Detection of cell proliferation with cell counting kit-8 (CCK-8) assay

Cells were incubated in serum-free medium for 24 hours and cells in the logarithmic growth stage were obtained and centrifuged for 5 minutes at 64 × *g* at room temperature. The cell density was adjusted to 2×10^4 cells/mL, and the suspensions were seeded into 96-well plates at 100 μL/well. After overnight, the cells were treated with doxorubicin (Dox) or 5-fluorouracil (5Fu) for 24 hours, the untreated cells used as negative control. Then, 10 μL of CCK-8 detection solution (ab228554, Abcam) was added to each well and incubated at 37°C for 1.5 hours. The optical density (OD) value was read at 450 nm, and the cell survival rate was calculated as follows: (OD450 treated group/OD450 control group) × 100%.

Mass spectrometry (MS)

$S100A4^{WT}$ and $S100A4^{KO}$ BMDMs were stimulated with IL-4 (20 ng/mL). After 36 hours, the cells were washed with cold PBS twice and harvested by ice-cold lysis buffer (pH 8.5, 8 M urea in 100 mM Tris-HCl with protease inhibitor cocktail). The cell lysates were centrifuged at 15 000 *g* for 30 minutes at 4°C to remove cell debris. Supernatants were collected and sent to process with MS analysis by Applied Protein Technology (Shanghai, China). The filters were set as follows: peptides with cut-off of FDR = 0.01 and significance threshold $P < 0.05$ (with 95% confidence).

Supplementary Figure 1.

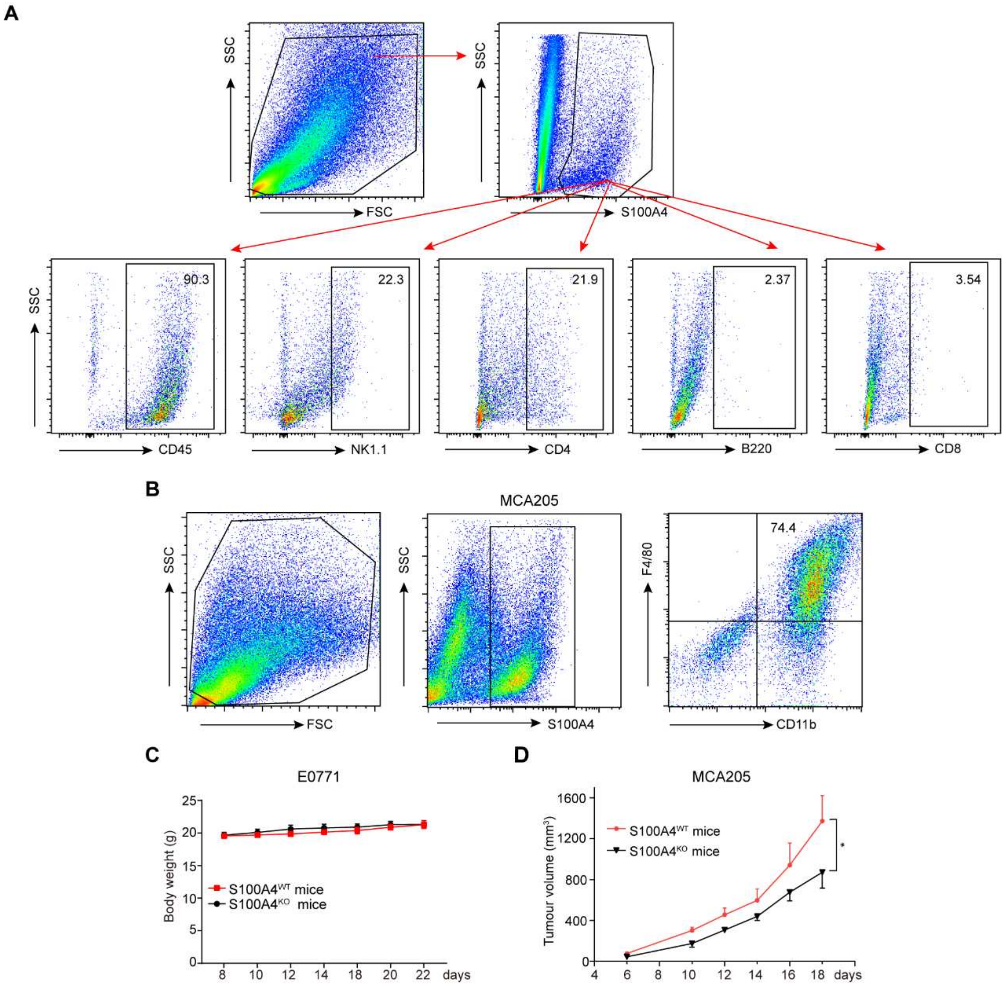

Supplementary Figure 1. Analysis of the immune contexture of tumour-infiltrating S100A4⁺ cells. (**A**) Flow-cytometry analysis of the frequencies of immune cells populations in tumour-infiltrating S100A4⁺ cells (upper) examined by staining with CD45, NK1.1, CD4, CD8 and B220 (lower). The tumour grafts are isolated from E0771 breast cancer cell-bearing S100A4$^{WT\text{-}EGFP}$ reporter mice. (**B**) Flow cytometric analysis of the frequencies of macrophages (stained with F4/80 and CD11b, right) in tumour-infiltrating S100A4⁺ cells isolated from MCA205 fibrosarcoma cell-bearing S100A4$^{WT\text{-}EGFP}$ reporter mice. (**C**) Body weight of E0771 breast cancer cell-bearing S100A4^{KO} or S100A4^{WT} mice (n≥5) was measured every 2 days after 7-days inoculation. (**D**) Growth of tumour grafts in tumour-bearing S100A4^{WT} or S100A4^{KO}

male mice (n≥5) was monitored over time after initial injection of MCA205 fibrosarcoma cells. Data are presented as mean ± s.e.m. and were analyzed with two-way ANOVA Sidak's multiple comparisons in **D**. The data are from one representative experiment of three independent experiments (**A-C**). **P* value<0.05.

Supplementary Figure 2.

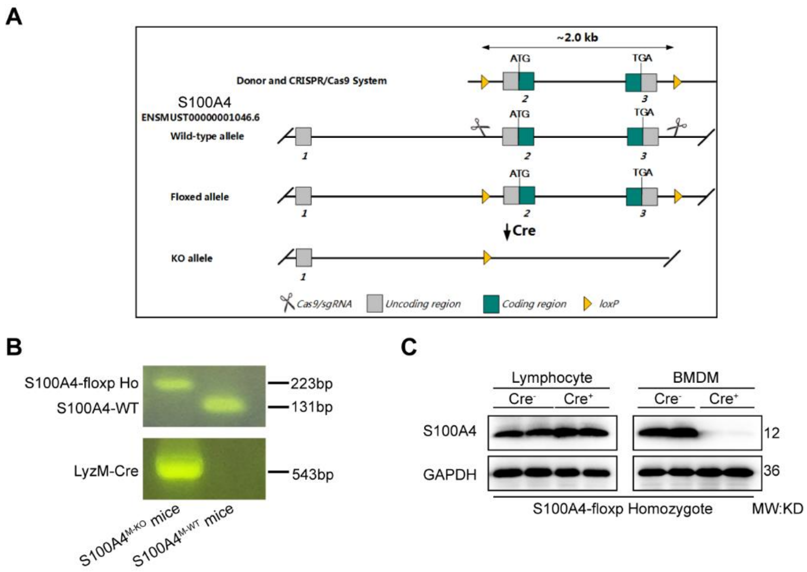

Supplementary Figure 2. Identifying the construction of the myeloid-deficient mouse. (**A**) Scheme of the construction of S100A4 floxp mouse. Targeted mice were crossed with WT mice to generate S100A4-floxed mice, which were further crossed with Lysosome M (LysM) Cre mice to generate myeloid cells conditional knockout mice. (**B**) Genotyping PCR analysis of S100A4$^{f/f}$LysM-cre^{-} and S100A4$^{f/f}$LysM-cre^{+} mice. (**C**) Immunoblotting analysis of S100A4 protein expression in macrophages and lymphocytes isolated from S100A4$^{M\text{-}WT}$ and S100A4$^{M\text{-}KO}$ mice. The data are from one representative experiment of three independent experiments (**B-C**).

Macrophagic S100A4 enhances protumour macrophage polarization

Supplementary Figure 3

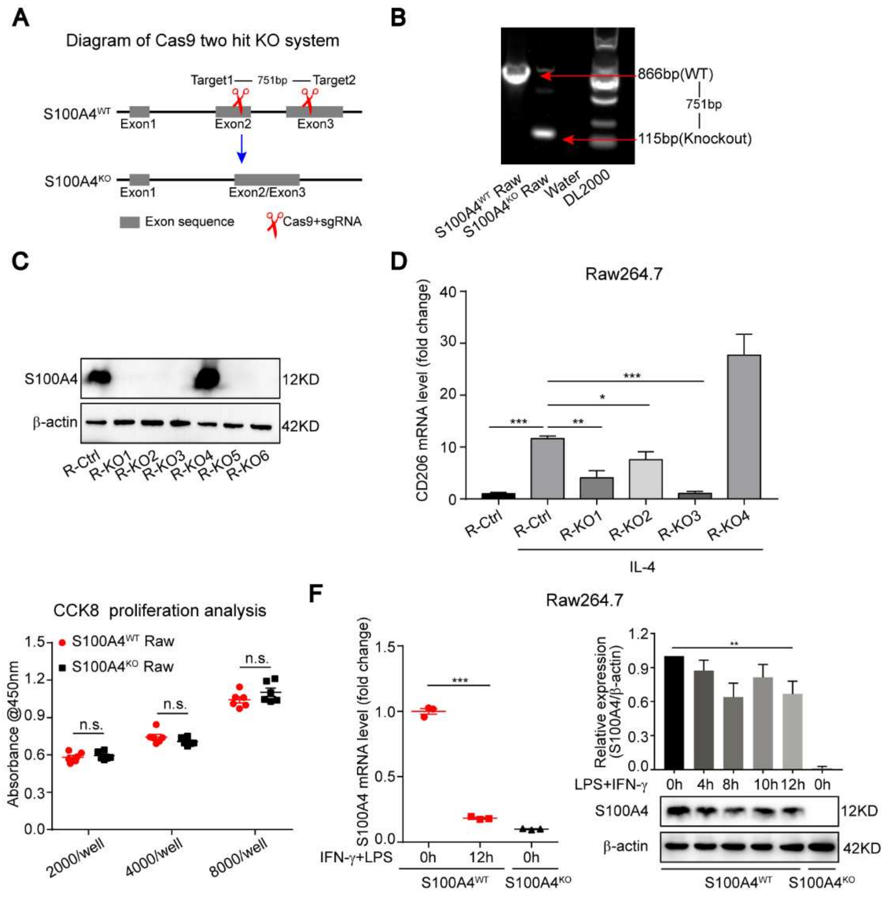

Supplementary Figure 3. Identifying the construction of S100A4 deficient Raw264.7 cell line. (**A-C**) Scheme of the construction of S100A4^{KO} Raw264.7 cell line. Mouse *s100a4* gene showing the CRISPR/Cas9-target sites. Individual exons are shown as boxes in **A**. Homologous recombination allele identification by PCR and sequencing in **B**. Screening positive colonies of S100A4^{KO} Raw264.7 cell by immunoblotting analysis in **C**. (**D**) The expression of protumour marker CD206 were detected by q-PCR in four S100A4 knockout monoclonal cell lines stimulated with IL-4 for 36 hours. (**E**) Proliferation assay in S100A4^{WT} or S100A4^{KO} Raw264.7 cells using CCK8 assays under indicated cell density in. Quantification of cell proliferation data collected from three independent experiments. (**F**) S100A4WT or S100A4KO Raw264.7 cells were activated IFN-γ (20 ng/mL) in combination

Liu S, *et al*. *J Immunother Cancer* 2021; 9:e002548. doi: 10.1136/jitc-2021-002548

with LPS (100 ng/mL). Nonactivated macrophages were used as controls. Expression of S100A4 mRNA and protein was analysed by q-PCR (left) and immunoblotting (right), respectively. Band densities (mean ± s.e.m.) for S100A4 were measured in at least three independent immunoblots and normalised to those of β-actin (loading control). Data are presented as mean ± s.e.m. and were analyzed with one-way ANOVA with Tukey's multiple comparisons. The data are from one representative experiment of three independent experiments (**B-F**). ***P* value < 0.01, and n.s., not significant.

Supplementary Figure 4

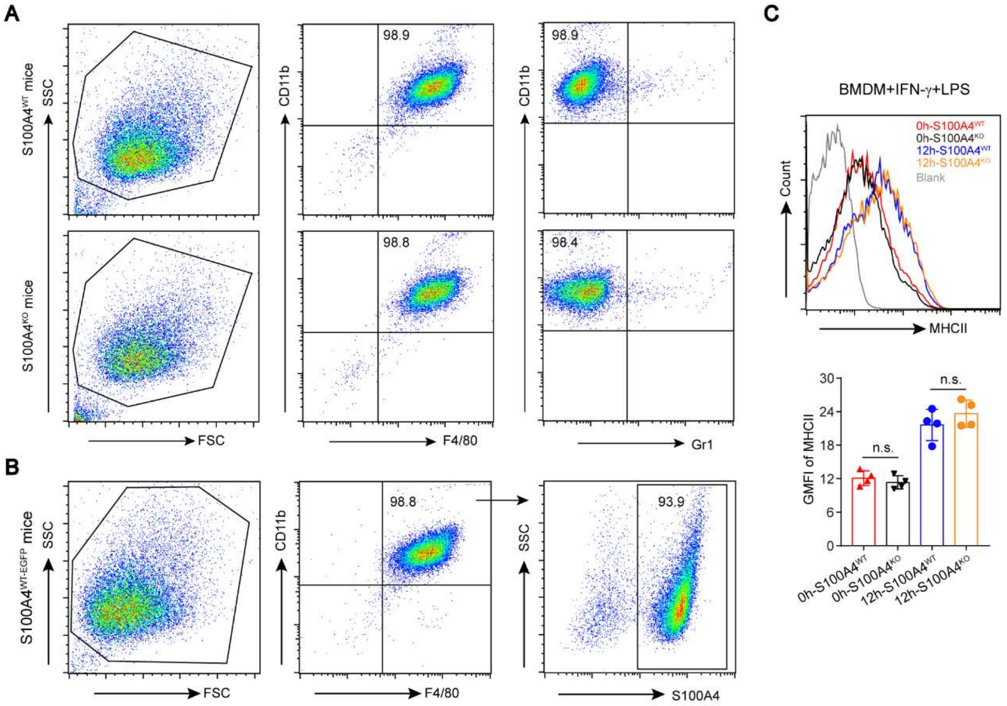

Supplementary Figure 4. Identifying the S100A4 expression in BMDMs. (**A**) Flow cytometric identification of BMDMs isolated from S100A4^{WT} or S100A4^{KO} mice. (**B**) Flow cytometric analysis the proportion of S100A4^{+} cells in BMDMs isolated from S100A4$^{WT\text{-}EGFP}$ mice. GMFI, geometric mean fluorescence intensity. (**C**) BMDMs isolated from S100A4^{WT} or S100A4^{KO} mice were activated IFN-γ (20 ng/mL) in combination with LPS (100 ng/mL).

Liu S, *et al. J Immunother Cancer* 2021; 9:e002548. doi: 10.1136/jitc-2021-002548

Macrophagic S100A4 enhances protumour macrophage polarization

Nonactivated macrophages were used as controls. Flow cytometric analysis of the expression of MHCII after stimulation with IFN-γ/LPS for 0 or 12 hours. GMFI, geometric mean fluorescence intensity. Data are presented as mean ± s.e.m. and were analysed by unpaired Student's *t*-test. The data are from one representative experiment of three independent experiments (**A-C**). **P* value < 0.05, ***P* value < 0.01, ****P* value < 0.001, n.s., not significant.

Supplementary Figure 5

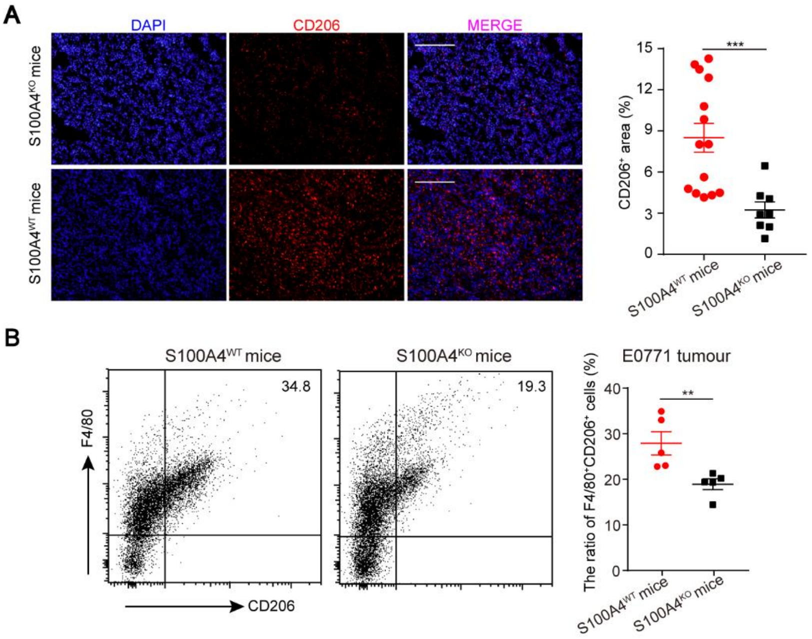

Supplementary Figure 5. S100A4 deficiency caused a decrease of CD206 expression. (**A**) Sections of carcinoma grafts from MCA205 fibrosarcoma cell-bearing S100A4^{WT} or S100A4^{KO} male mice were analyzed by immunofluorescence using specific antibody to CD206 (middle). The immune complexes were detected with a secondary antibody conjugated to Alexa Fluor 555 (red). DNA was stained with DAPI (blue). Scale bars, 200 μm. Immunofluorescent signal corresponding to CD206 was quantitated with Image J. (**B**) Flow cytometric analysis of the proportion of CD206^{+} TAMs in the tumour grafts from E0771 breast cancer cell–bearing S100A4^{WT} or S100A4^{KO} female mice. Data are presented as mean ± s.e.m and were analyzed with unpaired nonparametric Mann Whitney test. The data are from one

Liu S, *et al. J Immunother Cancer* 2021; 9:e002548. doi: 10.1136/jitc-2021-002548

representative experiment of three independent experiments (**A, B**). ***P value < 0.001.

Supplementary Figure 6.

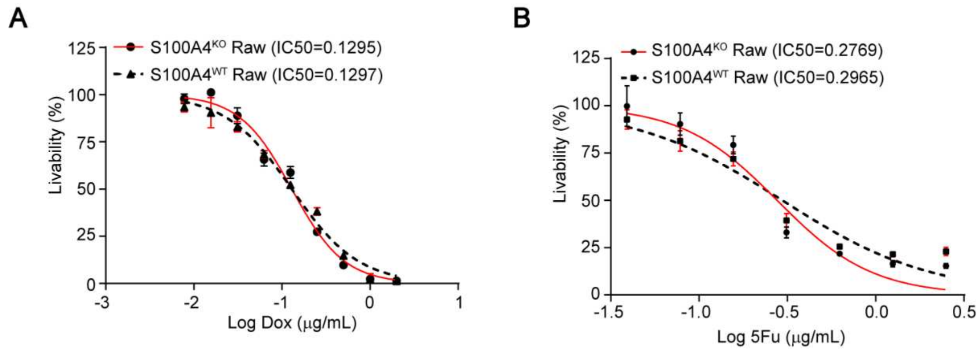

Supplementary Figure 6. S100A4 deficiency leads to decreased tumour angiogenesis. (**A, B**) Cell livability of S100A4^{WT} or S100A4^{KO} Raw264.7 cells, treated with doxorubicin (Dox in **A**) or 5-fluorouracil (5Fu in **B**) for 24 hours, was determined by CCK8 assay. Data are presented as mean ± s.e.m and were analyzed with unpaired nonparametric Mann Whitney test. The data are from one representative experiment of three independent experiments (**A, B**). **P value < 0.01.

Macrophagic S100A4 enhances protumour macrophage polarization

Supplementary Figure 7.

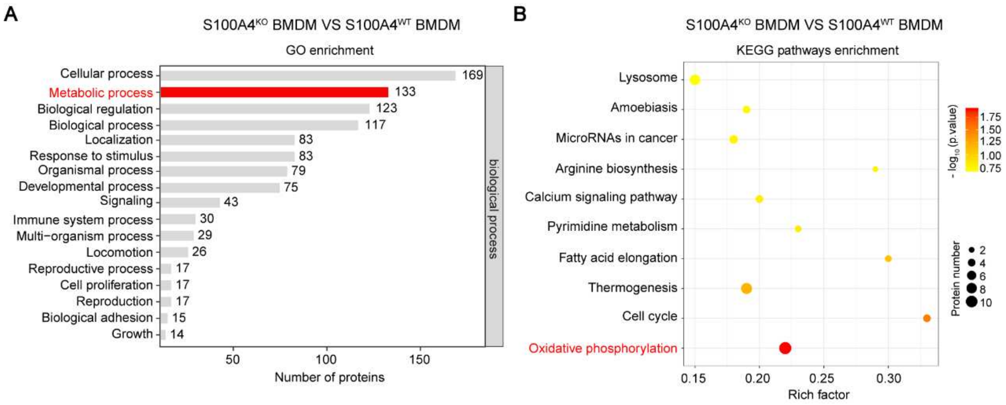

Supplementary Figure 7. MS analysis of the significantly changed pathways between $S100A4^{WT}$ and $S100A4^{KO}$ BMDMs. (**A**, **B**) Mass spectrometry (MS) analysis of the significantly changed peptides between $S100A4^{WT}$ and $S100A4^{KO}$ BMDMs after 36-hours stimulation with IL4 (20 ng/mL). Gene ontology (GO) term was shown in **A**, corrected P-value is large than 0.95. KEGG analysis of differential signaling pathways were shown in **B**.

Liu S, *et al. J Immunother Cancer* 2021; 9:e002548. doi: 10.1136/jitc-2021-002548

Macrophagic S100A4 enhances protumour macrophage polarization

Supplementary Figure 8.

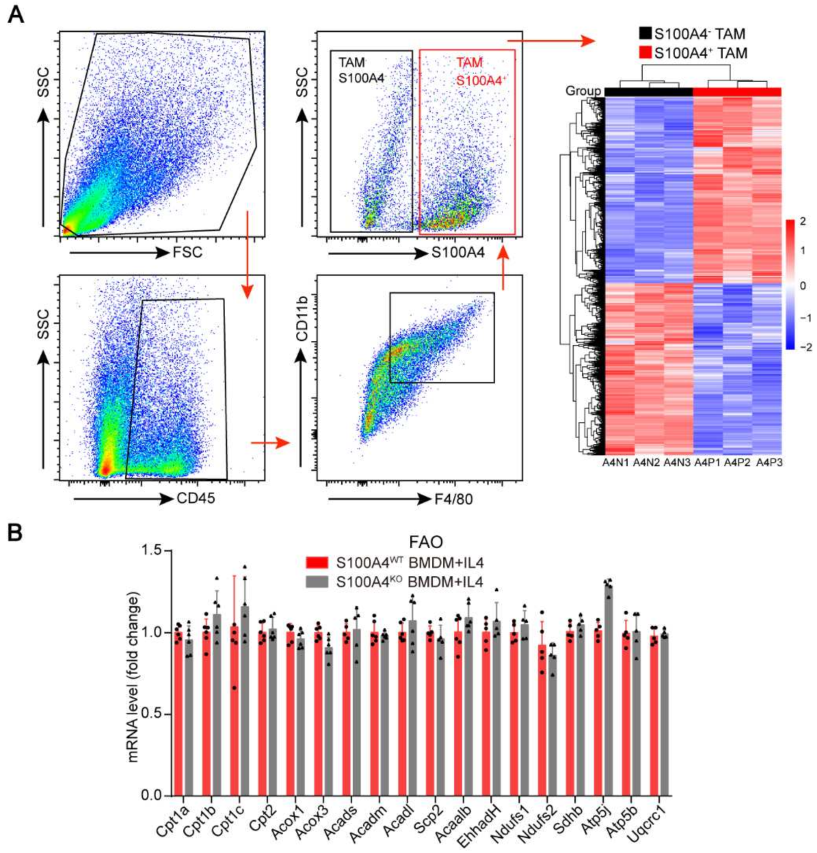

Supplementary Figure 8. RNA-sequencing analysis of S100A4^{+} and S100A4$^{–}$ TAMs. (**A**) Murine TAMs that isolated from the E0771 breast cancer cell-bearing S100A4$^{WT\text{-}EGFP}$ mice were sorted by flow cytometry into two subset populations: CD45^{+} F4/80^{+} CD11b^{+} EGFP^{+} and CD45^{+} F4/80^{+} CD11b^{+} EGFP$^{–}$ (left panel). The sorted cells were applied for total RNA extraction and subjected to RNA-sequencing analysis (right panel). (**B**) q-PCR analysis of gene expression in fatty acid oxidation of S100A4^{WT} and S100A4^{KO} BMDMs after 36 hours stimulation with IL4 (20 ng/mL).

Liu S, *et al. J Immunother Cancer* 2021; 9:e002548. doi: 10.1136/jitc-2021-002548

Macrophagic S100A4 enhances protumour macrophage polarization

Supplementary Figure 9

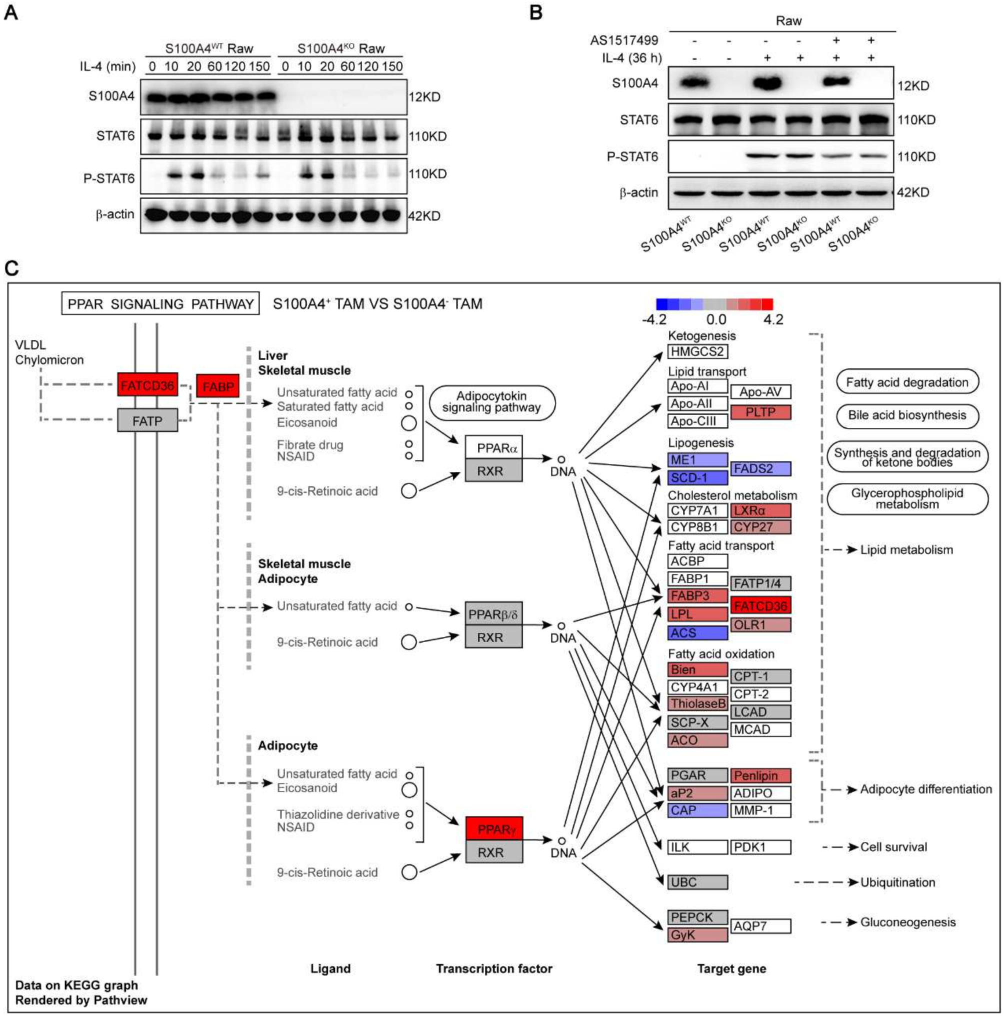

Supplementary Figure 9. Analysis of STAT6 and PPARs pathways in S100A4^{+} and S100A4$^{–}$ macrophages. (**A**) Immunoblotting analysis of the protein levels of S100A4, STAT6, and phospho-STAT6 in S100A4^{WT} or S100A4^{KO} Raw264.7 cells after stimulation with IL4 (20 ng/mL) for indicated times. (**B**) Immunoblotting analysis of the protein levels of S100A4, STAT6, and phospho-STAT6 in S100A4^{WT} or S100A4^{KO} Raw264.7 cells after stimulation with or without IL4 (20 ng/mL) and STAT6 inhibitor (AS1517499, 200 nM) for 36 h. (**C**) KEGG analysis of PPARs signaling pathways between the S100A4^{+} and S100A4^{-} TAMs isolated from E0771 breast cancer cell-bearing mice. The data are from one representative experiment of three independent experiments (**A, B**).

Macrophagic S100A4 enhances protumour macrophage polarization

Supplementary Figure 10

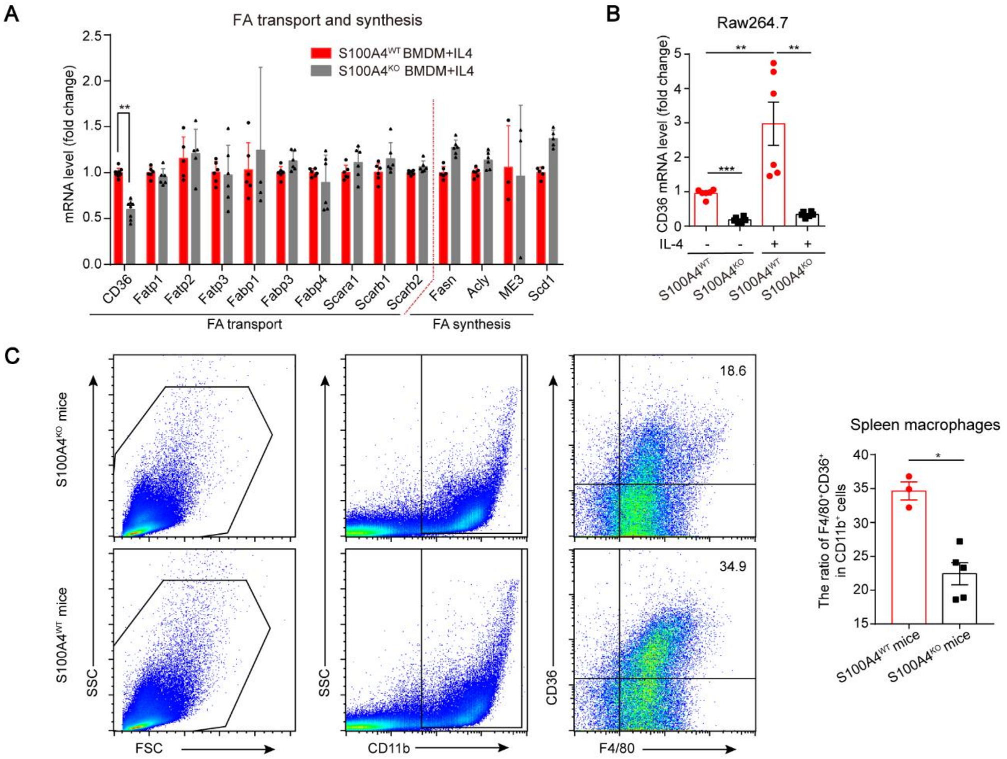

Supplementary Figure 10. S100A4 is required for CD36 upregulation in IL-4-activated macrophages. (**A**) q-PCR analysis of gene expression of fatty acid transport and synthesis in IL-4-activated S100A4^{WT} and S100A4^{KO} BMDMs. (**B**) q-PCR analysis of CD36 expression in IL-4-activated S100A4^{WT} and S100A4^{KO} Raw264.7 cells. In **A, and B**, cells were treated with IL-4 (20 ng/mL) for 36 hours. (**C**) Flow cytometric of the frequencies of CD36^{+} macrophages isolated from the spleen of S100A4^{WT} or S100A4^{KO} mice. Data are presented as mean ± s.e.m. and were analyzed with two-way ANOVA with Tukey's multiple comparisons in **A** and with unpaired nonparametric Mann Whitney test in **C**. The data are from one representative experiment of three independent experiments (**C**). *P value<0.05, **P value<0.01, ***P value<0.001.

Macrophagic S100A4 enhances protumour macrophage polarization

Supplementary Figure 11

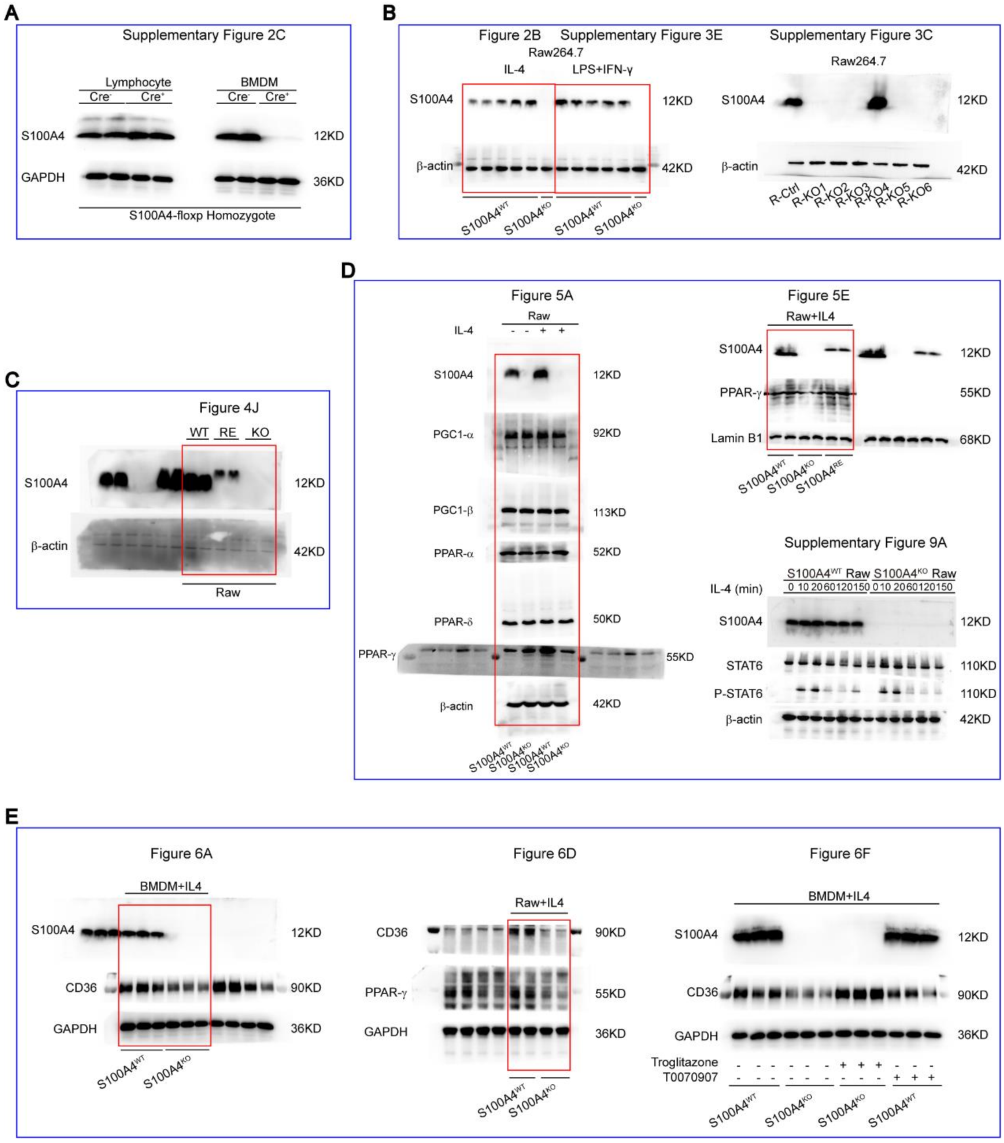

Supplementary Figure 11. All uncropped versions of the Western Blots. Images are presented in the same order as in the corresponding figures. The data are from one representative experiment of three independent experiments (**A-E**).

Liu S, *et al. J Immunother Cancer* 2021; 9:e002548. doi: 10.1136/jitc-2021-002548

Supplementary Figure 12

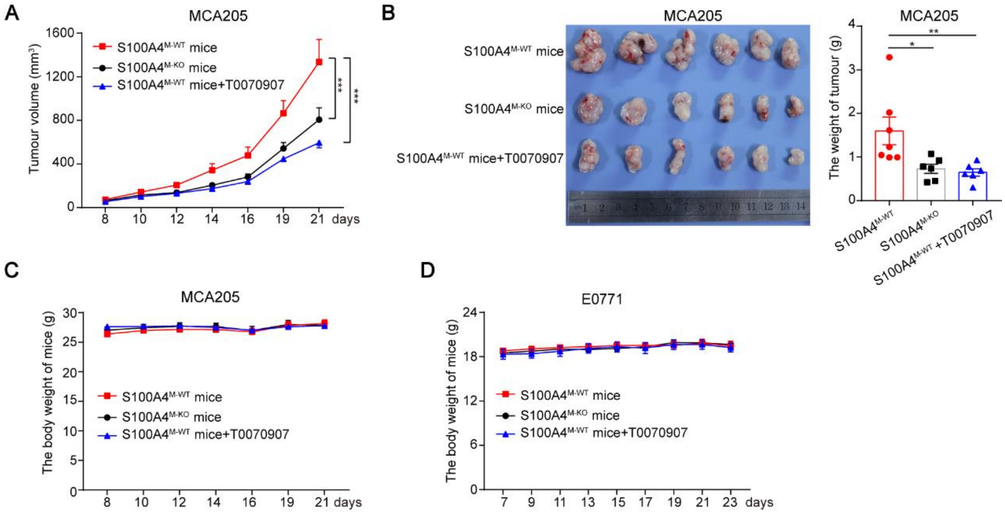

Supplementary Figure 12. S100A4 depletion and PPAR-γ inhibition have similar effects on intervention of TAM protumor polarization. (**A-C**) S100A4$^{M\text{-}WT}$ or S100A4$^{M\text{-}KO}$ male mice (n≥6) were implanted with MCA205 fibrosarcoma cells. Growth of tumour grafts (**A**) and mouse body weight (**C**) were monitored over time after the initial cell injection. Tumour weight (**B**) and representative pictures of tumour grafts excised at the end of the experiment are shown. (**D**) Body weight of E0771 breast cancer cell-bearing S100A4$^{M\text{-}WT}$ (with or without PPAR-γ inhibitor, T0070907) or S100A4$^{M\text{-}KO}$ female mice was measured every 2 days after 7-days inoculation. Data are presented as mean ± s.e.m. and were analyzed with two-way ANOVA with Dunnett's multiple comparisons in **A** or with Tukey's multiple comparisons **B.** **P* value<0.05, ***P* value<0.01, ****P* value<0.001.

Supplementary Table 1. The primers used in q-PCR

Genes	Forward	Reverse
Ndufs1	5'-AGG ATA TGT TCG CAC AAC TGG-3′	5'-TCA TGG TAA CAG AAT CGA GGG A-3′
Ndufs2	5'-CAG CCA GAT ATT GAA TGG GCA-3′	5'-TGT TGG TCA CCG CTT TTT CCT-3′
Sdhb	5'-AAT TTG CCA TTT ACC GAT GGG A-3′	5'-AGC ATC CAA CAC CAT AGG TCC-3′
Atp5j	5'-TAT TGG CCC AGA GTA TCA GCA-3′	5'-GGG GTT TGT CGA TGA CTT CAA AT-3′

Atp5b	5’-GGT TCA TCC TGC CAG AGA CTA-3′	5’-AAT CCC TCA TCG AAC TGG ACG-3′
Uqcrc1	5’-AGA CCC AGG TCA GCA TCT TG-3′	5’-GCC GAT TCT TTG TTC CCT TGA-3′
GAPDH	5’-AGG TCG GTG TGA ACG GAT TTG-3′	5’-TGT AGA CCA TGT AGT TGA GGT CA-3′
CPT2	5’-CAG CAC AGC ATC GTA CCC A-3′	5’-TCC CAA TGC CGT TCT CAA AAT-3′
Acox1	5’-CTC TCT ATG GGA TCA GCC AGA A-3′	5’-CCA CTC AAA CAA GTT TTC ATA CAC A-3′
Acox3	5’-TTG AGA AGA TCT ATA GCC TGG AGA TTT-3′	5’-AGT TCG GTG AGA GCA AAA CAG C-3′
Acads	5’-AGG TTA AGA AGA TGG GTG AGC TCG-3′	5’-ATG GAG TAG GCC AGG TAA TCC AAG-3′
Acadm	5’-AGG GTT TAG TTT TGA GTT GAC GG-3′	5’-CCC CGC TTT TGT CAT ATT CCG-3′
Acadl	5’-TCT TTT CCT CGG AGC ATG ACA-3′	5’-GAC CTC TCT ACT CAC TTC TCC AG-3′
Scp2	5’-CCT TCT GTC GCT TTG AAA TCT CC-3′	5’-GCT TCC TTT GCC ATA TCA GGA T-3′
Acaa1b	5’-TCT CCA GGA CGT GAG GCT AAA-3′	5’-CGC TCA GAA ATT GGG CGA TG-3′
Ehhadh	5’-ATG GCT GAG TAT CTG AGG CTG-3′	5’-GGT CCA AAC TAG CTT TCT GGA G-3′
ME3	5’-TCG ACC CGT ACC CCT GAA G-3′	5’-TCG CTC TGC TGA TTC TCA TAG T-3′
Scd1	5’-TTC TTG CGA TAC ACT CTG GTG C	5’-CGG GAT TGA ATG TTC TTG TCG T-3′
Fads2	5’-AAG GGA GGT AAC CAG GGA GAG-3′	5’-CCG CTG GGA CCA TTT GGT AA-3′
Fatp1	5’-GGC AAG CTC CAG CAC AGG AT-3′	5’-GTC CAC GGA AGT CCC AGA AAC-3′
Fatp2	5’-CAT CGT GGT TGG GGC TAC TT-3′	5’-GGT ACC GAA GCA GTT CAC CA-3′
Fatp3	5’-CGT GCT GGC CAC AGA GTT-3′	5’-ATT AGT TTC AGG GCC CGT CG-3′
Acly	5’-GCC AGC GGG AGC ACA TC-3′	5’-CTT TGC AGG TGC CAC TTC ATC-3′
Dgat1	5’-TAG AAG AGG ACG AGG TGC GA-3′	5’-GTC TTT GTC CCG GGT ATG GG-3′
Dgat2	5’-ACT GGA ACA CGC CCA AGA AA-3′	5’-GTA GTC TCG GAA GTA GCG CC-3′
Cpt1a	5’-ACG GAG TCC TGC AAC TTT GT-3′	5’-GTA CAG GTG CTG GTG CTT TTC-3′

Macrophagic S100A4 enhances protumour macrophage polarization

Cpt1b	5’-CCA ATC ATC TGG GTG CTG G-3′	5’-AAG AGA CCC CGT AGC CAT CA-3′
Cpt1c	5’-GGC TGG CAT TGG TCA GAA TC-3′	5’-CGT GCA ACC TCA GGA AGT C-3′
Fasn	5’-GGA GGT GGT GAT AGC CGG TAT-3′	5’-TGG GTA ATC CAT AGA GCC CAG-3′
Scara1	5’-GTG CTG TCT TCT TTA CCA GCA A-3′	5’-GCT GTC ATT GAA CGT GCG TC-3′
Scarb1	5’-TGA TGG AGA GCA AGC CTG TG-3′	5’-AGG ATC TCA CCA ACT GTG CG-3′
Scarb2	5’-CCT GCT CAG GGA GCT TAT CG-3′	5’-GTT CGT GCA CGG TGT GAA TC-3′
Fabp1	5’-AGG GGG TGT CAG AAA TCG TG-3′	5’-GTC ATG GTC TCC AGT TCG CA-3′
Fabp3	5’-GAC GGA GGC AAA CTC ATC CA-3′	5’-CAC CAC ACT GCC ATG AGT GA-3′
Fabp4	5’-CAT AAC CCT AGA TGG CGG GG-3′	5’-CCA GCT TGT CAC CAT CTC GT-3′
PPARγ	5’-CCC AAT GGT TGC TGA TTA CA-3′	5’-GGA CGC AGG CTC TAC TTT GA-3′
CD36	5’-ATG GGC TGT GAT CGG AACT G-3′	5’-GTC TTC CCA ATA AGC ATG TCT CC
CD206	5’-CAA GGA AGG TTG GCA TTT GT-3′	5’-CCT TTC AGT CCT TTG CAA GC-3′
Arg-1	5’-AAG AAT GGA AGA GTC AGT GTG G-3′	5’-GGG AGT GTT GAT GTC AGT GTG-3′
TGF-β	5’-CTC CCG TGG CTT CTA GTG C-3′	5’-GCC TTA GTT TGG ACA GGA TCT G-3′
S100A4	5’-TCA GCA CTT CCT CTC TCT TGG TCT G-3′	5’-TCC CCA GGA AGC TAG GCA GCT C-3′
PD-L1	5’-GCT CCA AAG GAC TTG TAC GTG-3′	5’-TGA TCT GAA GGG CAG CAT TTC-3′
Mgl2	5’-GGC CTC CAA TTC TTG AAA CCT-3′	5’-TTA GCC AAT GTG CTT AGC TGG-3′

Liu S, *et al. J Immunother Cancer* 2021; 9:e002548. doi: 10.1136/jitc-2021-002548
