# Supplementary material for: S100A4 enhances protumor macrophage polarization by control of PPAR-γ-dependent induction of fatty acid oxidation
Source: J Immunother Cancer. 2021 Jun 18;9(6):e002548. doi: 10.1136/jitc-2021-002548 (PMC8215236; doi:10.1136/jitc-2021-002548)

S100A4 enhances protumour macrophage polarization by control of PPAR-γ-dependent induction of fatty acid oxidation

Authors

Shuangqing Liu, Huilei Zhang, Yanan Li, Yana Zhang, Yangyang Bian, Yanqiong Zeng, Xiaohan Yao, Jiajia Wan, Xu Chen, Jianru Li, Zhaoqing Wang, and Zhihai Qin

Correspondence
zhihai@ibp.ac.cn
wangzq@ibp.ac.cn

In brief
Tumour-associated macrophages (TAMs) contained two subsets based on whether they expressed S100A4 or not.
S100A4 positive TAMs displayed protumour phenotypes with upregulated fatty acid oxidation.
Mechanistically, S100A4 enhances TAM protumour polarization by control of PPAR-γ-dependent induction of fatty acid oxidation

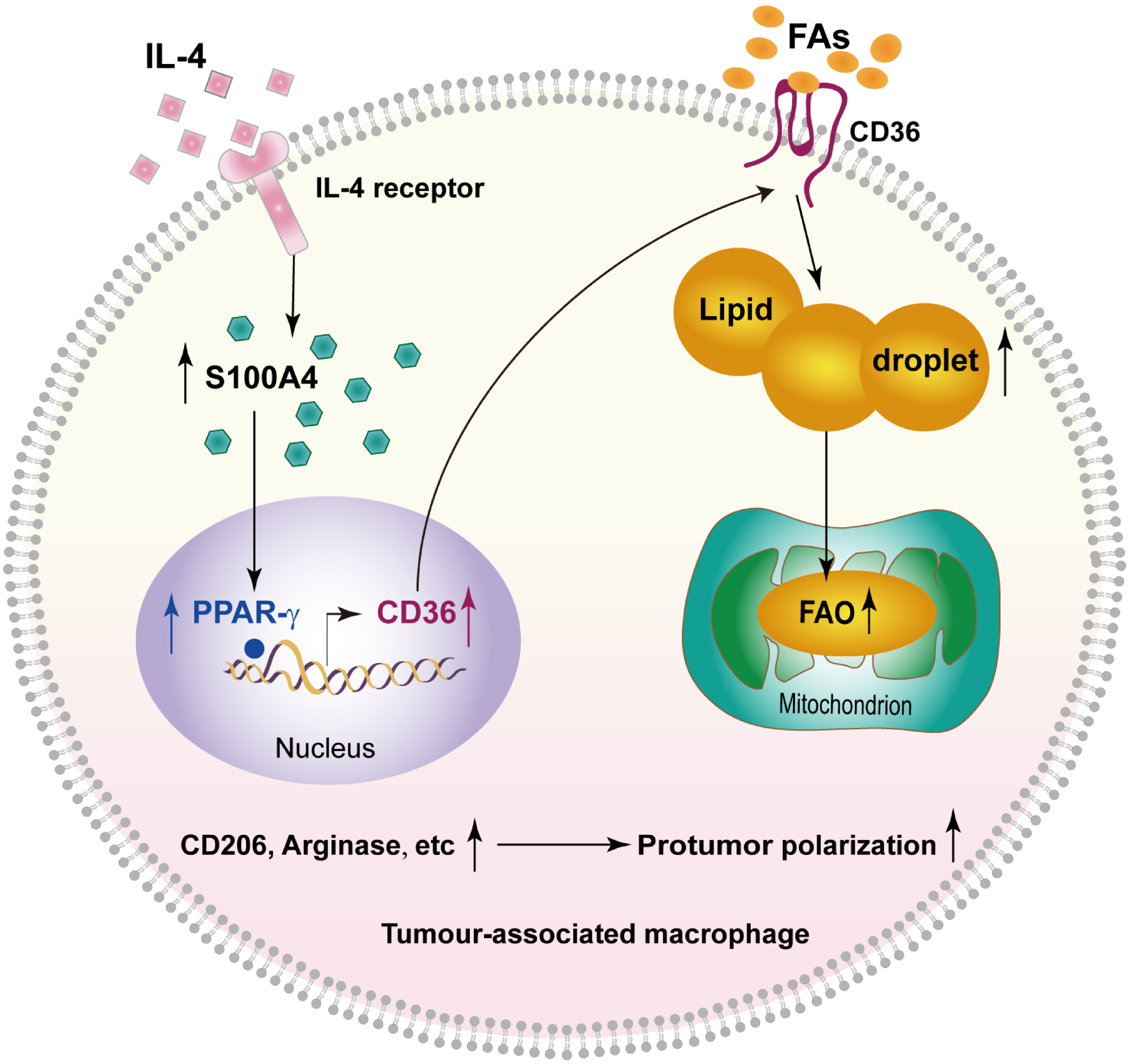

Supplement: Supplementary data [file jitc-2021-002548supp002.pdf]
